# Sertoli cells as a hub in testicular development and male reproductive

**DOI:** 10.3389/fcell.2025.1693878

**Published:** 2025-12-10

**Authors:** Xianzhou Feng, Jiahui Tian, Shuaiqi Han, Fei Wen, Yu Li, Shuaihang Zhang, Lidi Ouyang, Zhangtao Hu, Xiaoxu Chen, Jianhong Hu

**Affiliations:** 1 Key Laboratory of Animal Genetics, Breeding and Reproduction of Shaanxi Province, College of Animal Science and Technology, Northwest A&F University, Xianyang, Shaanxi, China; 2 Shaanxi Agriculture and Forestry Vocational and Technical University, Xianyang, Shaanxi, China; 3 Key Laboratory for Efficient Ruminant Breeding Technology of Higher Education Institutions in Shaanxi Province, Yangling, The Youth Innovation Team of Shaanxi Universities, Xianyang, Shaanxi, China

**Keywords:** testis, Sertoli cells, gonadal development, spermatogenesis, testosterone synthesis, testis immunity

## Abstract

In the early stages of mammalian embryonic development, the bipotential gonads can differentiate into the testes or ovaries. These organs are essential for gamete production, transmitting genetic information to offspring via sperm or oocytes. Testis differentiation is triggered by the Y chromosome sex-determining region (*SRY*) genetic program, with male reproductive health largely established during the early stages of testis development. However, *SRY* is only transiently activated in precursor Sertoli cells, initiating their differentiation. At later stages, differentiated Sertoli cells are crucial for male sex determining in other cell lineages, including germ cells, Leydig cells involved in steroid hormone synthesis, and the establishment of vascular patterns. Clearly, Sertoli cells play an essential role in testis development and function, and are indispensable for male reproduction. In this review, we examine the composition and functional dynamics of the testis, highlighting how single-cell transcriptomics has redefined our understanding of testicular cellular architecture and functional diversity. We focus on the pivotal regulatory roles of Sertoli cells in orchestrating the development and functional coordination of germ cells, Leydig cells, peritubular myoid cells, and testicular macrophages. Furthermore, we discuss the pathological consequences of Sertoli cell dysfunction and its mechanistic contributions to male reproductive disorders, providing molecular insights into spermatogenic failure and androgen dysregulation.

## 1 Introduction

**FIGURE 1:**
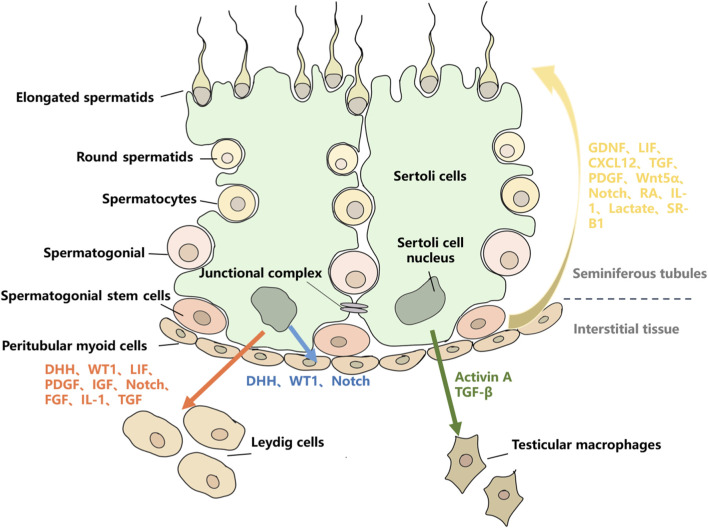
The regulatory roles of SCs on other testicular cell types. Red arrows represent the regulatory effects of SCs on LCs, mainly through the secretion of DHH, WT1, LIF, PDGF, IGF, Notch, FGF, IL-1, and TGF. Blue arrows represent the regulatory effects of SCs on PMCs, mainly through the secretion of DHH, WT1, and Notch. Green arrows represent the regulatory effects of SCs on TMs, mainly through the secretion of Activin-A and TGF-β. Yellow arrows represent the regulatory effects of SCs on GCs, mainly through the secretion of GDNF, LIF, CXCL12, TGF, PDGF, Wnt5α, Notch, RA, IL-1, Lactate, and SR-B1.

The testis is the primary organ of male sexual development and reproduction, secreting androgens to promote body masculinization and producing sperm for reproduction (Jiménez et al., 2021; Luo et al., 2022). Sertoli cells (SCs), as essential somatic cells in the testis, play a central role in testis formation during fetal development, as well as in the initiation and maintenance of spermatogenesis during puberty and adulthood. SCs also establish an immunoprivileged environment for germ cells (GCs), preventing autoimmune responses triggered by neoantigens during spermatogenesis (Luaces et al., 2023). Furthermore, SCs are key regulators of cell lineage differentiation within the testis, which promote male gonadal development (Rotgers et al., 2018), and specifies Leydig cells (LCs) and peritubular myoid cells (PMCs) fate (Li et al., 2016; Zhao et al., 2021). Postnatally, SCs also play a significant role in androgen production by LCs, development of PMCs, and regulation of the vasculature. Disruption of SCs function can lead to male reproductive disorders (see Figure 1). In this review, we systematically describe the central role of SCs in the testis, explore the mechanisms underlying their pathological dysfunction, and highlight how SCs serve as key factors in testicular development and functional maintenance. We hope this review offers insights into testis function and male reproductive health, encouraging further detailed investigations.

## 2 Composition and function of testis

**FIGURE 2:**
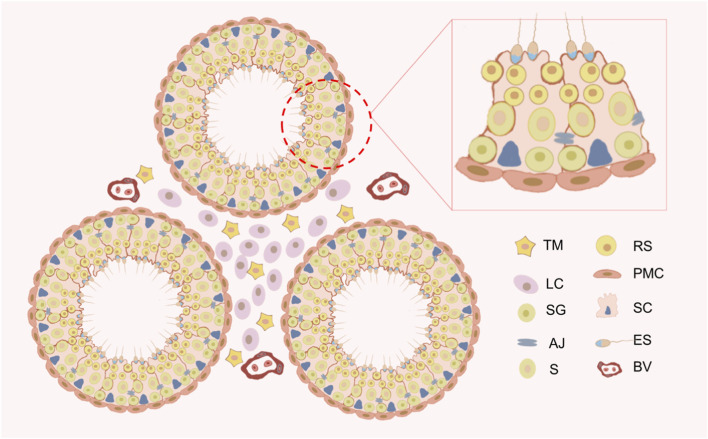
Schematic diagram of the testicular microenvironment of the mouse. The testis comprises two main components: the seminiferous tubules and the interstitial spaces. Peritubular myoid cells (PMCs) and Sertoli cells (SCs) together form the basal lamina of the seminiferous tubules. Germ cells develop in the seminiferous epithelium formed by SCs. Germ cells include spermatogonium (SG), spermatocytes (S), round spermatids (RS), and elongated spermatids (ES). The interstitial spaces primarily consist of Leydig cells (LCs) and testicular macrophages (TMs). Blood vessels (BV) are located within the interstitial spaces.

The testis comprises various types of cells, including GCs, SCs, LCs, PMCs, and testicular macrophages (TMs) (Mäkelä et al., 2019). Its tissue structure is primarily divided into two regions: the seminiferous tubules and the interstitial tissue (Oatley and Brinster, 2012). In the seminiferous tubules, SCs and PMCs form the basal lamina. GCs are located on the basal membrane and are classified into three types: spermatogonial stem cells (SSCs, undifferentiated spermatogonia), spermatogonia (SSCs that undergo active mitosis), and preleptotene spermatocytes (differentiated spermatogonia) (Wanjari and Gopalakrishnan, 2024). SCs support normal spermatogenesis by providing a cellular matrix and secreting specific growth factors (Chojnacka et al., 2016; Luca et al., 2018a). During spermatogenesis, SCs exhibit significant dynamic changes and are capable of supporting the simultaneous development of various types of GCs (Sawaied et al., 2025). The types and numbers of GCs supported by SCs vary at different stages of the spermatogenic cycle, typically including one to two types of spermatogonia, one to two types of spermatocytes, and one to two types of haploid spermatids. The number of GCs supported by SCs is regulated by various factors, primarily including species differences, the developmental status of SCs before puberty, SCs density within the seminiferous tubules, and the endocrine environment. In the mouse, spermatogenesis is divided into 12 stages, forming a complete spermatogenic cycle (O’Donnell et al., 2022a). In rodents such as mice, the spermatogenic cycle occurs sequentially along the length of the tubules, while in humans and primates, multiple spermatogenic cycles are interwoven, forming a more complex spermatogenic structure (O’Donnell et al., 2022b). The interstitial space contains LCs, mesenchymal cells, and immune cells (Oatley and Brinster, 2012). LCs produce androgens and cytokines, which may act directly or indirectly to regulate SSCs self-renewal (Zaker et al., 2022). GCs, Somatic cells, and various cytokines synthesized and secreted within the testis together form the testicular microenvironment (see Figure 2) (Zhou et al., 2019). Studies have shown that within the microenvironment, different types of cells communicate through cytokines to promote spermatogenesis and testosterone synthesis (Arora et al., 2022; Li et al., 2023). These interactions are essential for the normal functioning of the testis, including cell proliferation, differentiation, metabolism, and protein transport (Arora et al., 2022). SCs are the most critical somatic cells in testis, closely associated with GCs, supporting and guiding their development into mature spermatozoa during spermatogenesis (Griswold, 2018). Similarly, SCs play a vital role in maintaining the stability of SSCs pool (Ibtisham et al., 2020).

### 2.1 SCs

SCs, also known as “nanny cells”, are among the most intricate cells in the testis (França et al., 2016) and play crucial roles in protecting spermatogenic cells, providing nutrients and physical support for spermatogenesis and testis development (Griswold, 2018). SCs are classified based on stages into two types: immature Sertoli cells (ISCs, from fetal to pre-pubertal) and adult Sertoli cells (ASCs, from pubertal and post-pubertal) (You et al., 2021). ISCs participate in fetal sexual differentiation and pre-pubertal testicular development (Sekido and Lovell-Badge, 2013). Studies have shown that at E10-11 of mouse embryonic development, coelomic epithelial cells differentiate into gonadal somatic cells, which are bipotential can develop into either an ovary or a testis (Poulsen et al., 2019). The transition from the ovarian to the testis pathway is driven by the *SRY* gene expressed in pre-SCs during the narrow window of embryonic development (E10.5) (Hiramatsu et al., 2009; Bunce et al., 2023). By E12.5 of embryonic development, SCs form seminiferous cords around gonocytes (Yokonishi and Capel, 2021). Fetal Sertoli cells (FSCs) proliferation is essential for the lengthening and expansion of testicular seminiferous cords (Archambeault and Yao, 2010). After birth, SCs continue to proliferate until puberty, at which point they become non-proliferative and terminally differentiated (Meroni et al., 2019). ASCs maintain spermatogenesis during puberty and adulthood (Griswold, 2018) and provide immune protection for spermatogenic cells (Kaur et al., 2014). Normal SCs function is essential for GCs survival and fertility, and SCs dysfunction can lead to testicular pathology and male sterility (Murphy and Richburg, 2014; Ni et al., 2020). SCs exhibit strong secretory function, releasing various of substances, such as growth factors (including GDNF (Garcia et al., 2017), TGFs (Fang et al., 2023), NFG (Niu et al., 2020), SCF (Chui et al., 2011), ILs (Fadlalla, 2019), IGFs (Cannarella et al., 2019), FGFs (Tian et al., 2019), Activin (O’Donnell et al., 2022a)), transport protein (such as transferrin (Yefimova et al., 2008), androgen-binding proteins (Yefimova et al., 2008), sulfated glycoprotein (Hartmann et al., 2016), and ceruloplasmin-like protein proteins (Skinner and Griswold, 1983)) and regulatory proteins (AMH (Rodriguez et al., 2022), endothelin (Chan et al., 2008), and inhibin (Lucas-Herald and Mitchell, 2022)), these substances regulate the self-renewal and differentiation of spermatogenic cells, as well as the growth and development of the male reproductive system.

### 2.2 LCs

**FIGURE 3:**
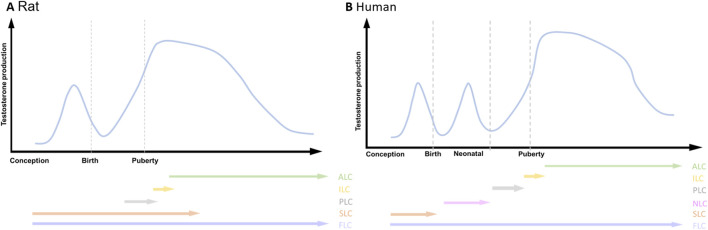
Testosterone production and the emergence of LCs during the life span. **(A)** Rat; **(B)** Human. adult Leydig cells (ALC), immature Leydig cells (ILC), progenitor Leydig cells (PLC), neonatal Leydig cells (NLC), stem Leydig cells (SLC), fetal Leydig cells (FLC). Two androgenic peaks are present in Rat, while three androgenic peaks are present in human.

LCs are located in the interstitial spaces of the seminiferous tubules and are mainly responsible for androgen synthesis and secretion, essential for spermatogenesis (Fang et al., 2021; Medici et al., 2024). In mammals, at least two types of LCs exist: fetal Leydig cells (FLCs) and adult Leydig cells (ALCs). FLCs synthesize androgen in the testis before birth, while ALCs perform this function after birth (Teerds and Huhtaniemi, 2015; Shima, 2019). FLCs produce androstenedione, which requires converted to testosterone in the presence of FSCs (Shima et al., 2013). During embryonic development, FLCs and FSCs jointly produce high androgen levels, promoting male reproductive organ differentiation and brain masculinization (Lee et al., 2024). Postnatally, FLCs decrease in number, resulting in reduced androgen production, which eventually reaches a minimum. With the development of ALCs from testicular stem cells, androgen levels gradually increase (Chauvigné et al., 2012; Zirkin and Papadopoulos, 2018). Testosterone regulates mammalian spermatogenesis, and its deficiency in adults leads to reduced fertility, fat accumulation, impaired cognitive function, weakened immune response, and fatigue (Chen H et al., 2017). Rodent LCs development proceeds through four main stages: stem Leydig cells (SLCs), progenitor Leydig cells (PLCs), immature Leydig cells (ILCs), and ALCs, as illustrated in Figure 3A (Chen M et al., 2017). However, another type of LCs exists in the primate and human testis, short-lived during the neonatal stage, thought their origin remains unclear (Nistal et al., 1986; Prince, 1992; Prince, 2001). The hypothalamic-pituitary-testicular axis (HPTA) is transiently activated during the first 6 months after birth, promoting neonatal LCs development and increasing androgen levels. After this period, the HPTA returns to inactivity, and neonatal LCs either involute or dedifferentiate (as shown in Figure 3B) (Prince, 1990). During this brief period, the function of nascent LCs is largely unstudied and remains unknown. It is hypothesized that testosterone synthesis by neonatal LCs is linked to the central nervous system and contributes to brain development (Martin, 2016).

### 2.3 TMs

TMs represent the largest reservoir of immune cells in the mammalian testis, accounting for over 20% of interstitial cells (Hume et al., 1984; Masone, 2023). TMs preserve testicular immune privilege by modulating the innate immune response (Bhushan et al., 2015; Bhushan and Meinhardt, 2017), thus attenuating pro-inflammatory responses triggered by external stimuli (Meinhardt et al., 2018). TMs are categorized into two types: interstitial macrophages and peritubular macrophages, with the former originating from embryo yolk sac cells (DeFalco et al., 2014). Fetal TMs play a crucial role in testicular morphological changes during early gonadal development and regulate vascular remodeling (Li Q et al., 2021). After birth, bone marrow progenitor cells differentiated into peritubular macrophages, which colonize empty niches as specialized tissue-resident macrophages (Mossadegh-Keller et al., 2017). Cytokines secreted by TMs promote LCs proliferation and differentiation (Gaytan et al., 1994), and stimulate steroid hormone synthesis (Hales, 2002; Hutson, 2006). These factors also contribute to the maintenance and differentiation of SSCs (DeFalco et al., 2015; Mossadegh-Keller et al., 2017), which are essential for testicular development and spermatogenesis.

### 2.4 PMCs

PMCs are a major component of the seminiferous tubule wall, characterized by smooth muscle-like features and playing a key role in sperm transport (Wang et al., 2018a). Evidence suggests that PMCs are closely related to spermatogenesis. During embryonic development, PMCs and SCs together form the basal lamina of the seminiferous epithelium, creating a unique microenvironment for SSCs self-renew, which maintain the number of spermatogonia and spermatocytes in the testis (Richardson et al., 1995; Uchida et al., 2020; Min et al., 2022). PMCs secrete leucine-rich repeat-containing G protein-coupled receptor 4 (LGR4), colony-stimulating factor-1 (CSF-1), and glial cell-derived neurotrophic factor (GDNF), all of which regulate spermatogenesis and testicular function (Maekawa et al., 1996; Fernandez et al., 2008; Qian et al., 2013; Chen and Liu, 2016). However, when spermatogenesis is disrupted, the structure and function of PMCs undergo changes (Mayerhofer, 2013). Additionally, other cells in the TME secrete paracrine factors that can act on PMCs, promoting cell contraction, relaxation, and seminiferous tubule peristalsis (Fleck et al., 2021; Kawabe et al., 2022). Loss of PMCs contractile function results in sterility (Wang et al., 2018b).

### 2.5 GCs

**FIGURE 4:**
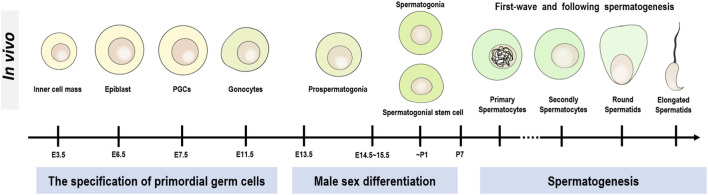
Development of GCs in male mice. The entire development of male mice GCs is divided into three main stages, the first being the specification of primordial germ cells (PGCs), the second being the stage of male sex differentiation, and the third being the stage of spermatogenesis.

In contrast to somatic cells in the testis, GCs play a crucial role in reproduction by accurately transmitting genetic information from one generation to the next (Yamashita, 2020). GCs comprise SSCs, spermatogonia, early spermatocytes, spermatids, and elongated spermatozoa (Hancock et al., 2021; Ren et al., 2022). In mammals, including mice and humans, male GCs development occurs in three main stages (see Figure 4): specification of primordial germ cells (PGCs), male sex differentiation, and spermatogenesis (Saitou and Miyauchi, 2016). PGCs are induced following the differentiation of trophectoderm and primitive endoderm (Saitou and Yamaji, 2012; Sasaki et al., 2016). PGCs, the earliest GCs in mice (appearing at E6.25), are detected around E7.25 (Richardson and Lehmann, 2010; Saitou and Yamaji, 2010). PGCs begin migrating at E7.5 and colonize the genital ridge at E10.5 (Seki et al., 2007; Nicholls and Page, 2021). Upon reaching the genital ridge, PGCs undergo mitotic arrest, remaining in the G0/G1 phase and becoming quiescent (Western et al., 2008). The first spermatogonia typically appear shortly after birth, and then the spermatogonia migrate from the center of the seminiferous tubules to the periphery, where they continue to proliferate (De Rooij and Russell, 2000; Nagano et al., 2000). In many male species (such as rats, mice, cats, sheep, donkeys, cattle), spermatogenesis occurs continuously, forming meiotic spermatocytes, spermatids, and ultimately elongated sperm. However, in humans and other primates, there is a long interval between the appearance of spermatogonia and the completion of spermatogenesis, which typically begins at puberty. Additionally, in seasonally breeding mammals, the first wave of spermatogenesis must occur at the start of each breeding season because a complete set of germ cells must be formed from spermatogonia (Jones et al., 2021). It has been reported that spermatogenesis begins on postnatal day 5 in mice, and a portion of the PGCs are recruited as SSCs, which migrate to the basement membrane of the seminiferous tubules and gradually move toward the lumen as their differentiation progresses (Oatley and Brinster, 2012; Yoshida, 2020). SSCs Self-renewal and differentiation maintain the stem cell pool, forming the foundation for sperm production (Walker, 2010). In rodents, A_single_ spermatogonium are considered SSCs. Through mitosis, A_single_ spermatogonium form two A_paired_ spermatogonia, if no intercellular bridges are present, they undergo self-renewal, producing two new A_single_ spermatogonium; if intercellular bridges form, they differentiate into A_aligned-4_, A_aligned-8_, and A_aligned-16_ (Dym et al., 2009). A_aligned-16_ differentiate into A1 spermatogonia, which give rise to A2, A3, A4, intermediate, and B spermatogonia through successive mitotic divisions, eventually forming primary spermatocytes (Wakayama et al., 2015). Primary spermatocytes undergo two meiotic divisions to form secondary spermatocytes and round spermatids, which then undergo spermatid metamorphosis to become elongated spermatozoa (Zhang L. et al., 2022). In mice, A_single_, A_pair_, and A_aligned_ are considered undifferentiated spermatogonia, with A_single_ accounting for 10% of this cell population (Nakagawa et al., 2007; Fayomi and Orwig, 2018). However, in primates, the classification of spermatogonia differs from that in mice. The spermatogonia of primates are classified into three types: A_dark_, A_pale_, and B. A_dark_ spermatogonia are considered reserve-type, while A_pale_ spermatogonia are proliferative-type, and B spermatogonia correspond to the differentiated cell population in mice (Lord and Oatley, 2017; Potter and DeFalco, 2017). Research indicates that A_dark_ spermatogonia are a form of quiescent SSCs that typically only proliferate during puberty or when a large number of spermatogonia are lost due to certain factors, in order to restore the self-renewal capacity of SSCs. A_pale_ spermatogonia constitute the majority of SSCs and undergo self-renewal and proliferation in a regulated manner according to the spermatogenic epithelial cycle (Kubota and Brinster, 2018). However, there is some debate surrounding this concept.

### 2.6 Single-cell transcriptomics redefines testicular cellular architecture and functional diversity

Spermatogenesis, the cornerstone of male reproduction, involves the proliferation and differentiation of diploid germ cells into haploid flagellated spermatozoa. This tightly orchestrated process relies on intricate interactions between testicular somatic cells (e.g., SCs and LCs) and GCs. However, its pronounced cellular heterogeneity has posed long-standing challenges in resolving stage-specific cellular populations. While the roles of somatic cells in spermatogenesis are relatively well-defined, the molecular mechanisms governing their developmental maturation remain incompletely elucidated.

The application of single-cell RNA sequencing (scRNA-seq) has systematically characterized the heterogeneity of mammalian testicular cells and redefined their cellular atlases. In yaks, pigs, humans, and other species, scRNA-seq has identified GCs subpopulations (spermatogonial stem cells, spermatocytes, and spermatids) and somatic subtypes (SCs, LCs, PMCs) with distinct markers (e.g., UCHL1, SOX9, PRND) (Wang et al., 2023a; Yan et al., 2024). Pseudotime trajectory analyses further indicate a shared progenitor origin for LCs and PMCs (Yan et al., 2024), and functional enrichment demonstrated synergistic regulation between somatic signaling pathways (PI3K-AKT, Wnt) and germline modules (spermatogenesis, DNA binding) (Yan et al., 2024). Cross-species comparisons show that these cell types exhibit both conserved functions and species-specific heterogeneity in pigs, humans, and mice (Zhang W. et al., 2022; Wang et al., 2024).

Compared with other testicular somatic cell types, the developmental trajectory of SCs has been extensively characterized by scRNA-seq. Most studies classify SCs into three stages: immature (during fetal/perinatal phase/neonatal), maturing (during juvenile/prepubertal life), and mature (at puberty and adulthood) (Guo et al., 2020; Tan et al., 2020; Zhao et al., 2020; Su et al., 2025). In human testes, SCs undergo three consecutive stages during normal maturation: Stage_a, b, and c, each with a distinct gene expression profile. Stage_a is characterized by high expression of *EGR3*, *JUN*, and *NR4A1*; Stage_b by *S100A13*, *ENO1*, and *BEX1*; and Stage_c cells primarily express *HOPX*, *DEFB119*, and *CST9L*. This trajectory shows marked age dependence. Stage_a cells are mainly SCs from 2 years old. The numbers of Stage_a and Stage_b cells decrease with age, reaching a minimum at 11 years. Thereafter, functionally mature Stage_c cells emerge and become predominant in late adolescence and adulthood. Functional enrichment analysis further supports the transition between developmental stages. Stage_a cells are significantly enriched for biological processes such as “stem cell differentiation” and “cell number maintenance”, indicating that SCs at this stage primarily exhibit characteristics of stem or progenitor cells. In contrast, Stage_c cells are enriched for processes including “compound rescue in cellular metabolism”, “protein transmembrane transport”, and “phagosome maturation”, indicating that mature SCs primarily function to phagocytose GCs and their metabolic byproducts (Zhao et al., 2020). Guo et al. also identified two immature SCs stages in human testes, designated Immature #1 and Immature #2, which ultimately merged into mature SCs at 11 years of age (Guo et al., 2020). Comparing the two studies, we observe that Immature one resembles Stage_a, while Immature two is closer to Stage_b. In testicular disease research, transcriptomic analyses comparing normal and Klinefelter syndrome (KS) testes indicate that SCs are the most severely affected somatic cell type (Winge et al., 2020). KS SCs are largely arrested at Stage_b and display altered energy metabolism features, such as upregulation of genes associated with oxidative phosphorylation and glycolysis, while triglyceride metabolism levels are suppressed (Zhao et al., 2020). They also show broad transcriptional dysregulation, including upregulation of immune-related genes (B2M and MIF (del Campo et al., 2014; Sumaiya et al., 2022)) and reduced expression of sex hormone-regulated gene *GNRH1* (Zhao et al., 2020). Notably, X-linked genes are overexpressed in KS SCs. Mahyari et al. provide a key explanation for this phenomenon: they identified a distinct subset of KS SCs lacking XIST expression, this mechanistic defect likely contributes to the upregulation of X-linked genes within KS SCs (Mahyari et al., 2021).

However, scRNA-seq’s reliance on tissue dissociation limits spatial insights into microenvironmental interactions. Recent advances in spatial transcriptomics now enable the characterization of molecular features and intercellular dynamics within native tissue contexts. These technologies map spatially variable genes, delineate cellular neighborhoods, reconstruct communication networks, and detect molecular alterations under pathological conditions (Xu and Chen, 2025). For instance, Slide-seq preserves spatial information to systematically unveil germ-somatic interaction networks (e.g., spermatogonial stem cells adjacent to SCs) within seminiferous tubules (Rajachandran et al., 2023). They also identify conserved (mouse-human) and disease-specific spatial gene modules (e.g., inflammatory signaling in diabetic models), providing a transformative framework for understanding testicular pathologies and therapeutic development (Chen et al., 2021).

## 3 Influence of SCs on gonadal development

### 3.1 SCs coordinate gonadal sex differentiation

Gonadal development is a complex process initiated by the migration of PGCs (Yildirim et al., 2020). During embryonic development, PGCs migrate alongside other cell types to the gonadal ridge, where they are induced to differentiate into ovaries or testes in response to different factors (Poulsen et al., 2019). In the early formation of the mouse genital ridge, coelomic epithelial cells express transcription factors WT1, SF1, GATA4, and LHX9, all of which are essential for the gonad formation (Birk et al., 2000; Wilhelm and Englert, 2002; Klattig et al., 2007; Chen H et al., 2017). The supporting cells are the first differentiated somatic cells, playing a pivotal role in orchestrating gonadal sex determination and promoting gametogenesis (Rotgers et al., 2018). These cells differentiate into SCs in the testis and granulosa cells in the ovary (Carré and Greenfield, 2016). This review focuses on the relationship between SCs and male gonadal development.

In mice, sex determination occurs shortly after the coelomic epithelium migrates into the gonadal ridge, simultaneously, the tunica albuginea forms at the junction between the gonad and the coelomic epithelium, preventing cell entry (Karl and Capel, 1998). Compared to other somatic lineages, SCs are almost entirely XY in chromosome composition of the XX-XY mouse chimera (Palmer and Burgoyne, 1991), that is: the SRY acts cell-autonomously in pre-SCs to trigger their differentiation, with further somatic masculinization resulting from SCs signaling. Karl and his colleagues’ research confirms this view, in the testis, SCs differentiate from the first wave of cells entering the gonads, while subsequent progenitor cells differentiate into LCs, PMCs, and TMs (Karl and Capel, 1998). Before differentiation, coelomic epithelial progenitor pools express both SF1 and WT1 (Liu et al., 2016). After differentiation, SCs retain WT1 expression, whereas LCs maintain higher levels of SF1 (Schmahl et al., 2000; Miyabayashi et al., 2013; McClelland et al., 2015). When the *WT1* gene is deleted in the gonadal primordium before sex determination, progenitor cells fail to activate the development programs for XX and XY gonads, leading instead to their differentiation into SF1-expressing steroidogenic cells (Chen M et al., 2017). One study indicates that WT1 influences progenitor cell fate towards SCs differentiation by reducing Nr5a1 transcription (Bale and Epperson, 2015).

Testis differentiation is regulated by the sex-determining gene *SRY* on the Y chromosome, which is expressed only in pre-SCs (Okashita and Tachibana, 2021). At the onset of sex determination, *SRY* genes are transiently upregulated in SCs (Del Valle et al., 2017). After sex determination, the *SRY* gene is silenced in mice, while in humans, there are lower levels of *SRY* gene expression persist even in mature testes (Hanley et al., 2000; Bullejos and Koopman, 2001). *SRY* gene expression is initiated by transcription factors, including SF1, WT1, GATA4, FOG2 and CBX2, in null mutant of these factors, the gonads exhibit reduced or missing *SRY* expression, resulting in XY sex reversal (Barbara et al., 2001; Hossain and Saunders, 2001; Tevosian et al., 2002; Miyamoto et al., 2008; Katoh-Fukui et al., 2012; Warr et al., 2012). As well, histone modifications, DNA methylation, and chromatin repression also regulate *SRY* expression (Kuroki and Tachibana, 2018). The SRY protein binds DNA, activating genes involved in testis development and repressing genes associated with ovary differentiation (Rotgers et al., 2018). The primary target gene of SRY is *SOX9*. SRY expression activates SOX9, which in turn activates FGF9, promoting the differentiation of pre-SCs (Sekido and Lovell-Badge, 2008; Zhao and Koopman, 2012). Once SCs are formed, they induce FLCs development through hedgehog signaling (Yao et al., 2002), promoting the production of testosterone and insulin-like 3 (INSL3) (Barsoum et al., 2009). This process further induces testicular descent and the masculinization of external genitalia (Hutson, 2013; Roth et al., 2021). In parallel, SOX9 recruits other proteins (PADI, AMH, and PDGS) to facilitate testis development (Otake and Park, 2016; Tsuji-Hosokawa et al., 2018; Lucas-Herald and Mitchell, 2022). However, the absence of *SRY* expression in pre-SCs leads to the differentiation of the gonad into ovaries (Albrecht and Eicher, 2001). Similarly, abnormal *SRY* expression causes the genital ridge to develop into ovaries (Larney et al., 2014). Therefore, *SRY* expressed by pre-SCs is essential for directing gonadal development towards masculinization.

### 3.2 SCs induce testis cords formation

After the gonadal primordium is established and the fate of the supporting cells is determined, the gonad develops into either ovaries or testes (Poulsen et al., 2019). Once specified for testis development, pre-SCs polarize, aggregate, and assemble around pre-GCs to form SC-GC clusters, then endothelial cells migrate and guide to form testis-specific tubular structures, known as testis cords, which later develop into seminiferous tubules in the adult testis (Rossi et al., 2000; Combes et al., 2009; Svingen and Koopmam, 2013). The maintenance of fetal testis cords is normal testis development (Archambeault and Yao, 2010; Chen et al., 2010; Cool et al., 2012; Zheng et al., 2015). The dramatic changes in testis cords morphology are driven by the rapid proliferation of FSCs, alterations in FSCs number influence the cord structures development, potentially leading to involution and the formation of blind-ending tubules (Bagheri-Fam et al., 2011). The initiation of cord formation requires a large number of FSCs to separate and enclose the GCs within the lumen (Brennan and Capel, 2004; Svingen and Koopman, 2013; Chen et al., 2016). Growing evidence suggests that TGFβ is associated with testis cord formation, though its effects may be indirect. TGFβ plays an important role in regulating and SCs proliferation. The specific deletion of Smad4 (a core component of TGFβ signaling) in SCs leads to defective proliferation and dysplastic testis cords (Archambeault and Yao, 2014). DNA damage binding protein 1 (DDB1) promotes SCs proliferation by activation of the TGFβ pathway, deletion of DDB1 in the FSCs impairs their proliferation and disrupts the coiling of testis cords, leading to smaller testes and reduced sperm production (Zheng et al., 2019). In addition, activin A, produced by FSCs, is a unique regulator of SCs proliferation and cords expansion (Archambeault and Yao, 2010). Taken together, FSCs play a significant role in promoting the elongation and expansion of cord structures, laying the foundation for postnatal testis development.

## 4 SCs promote spermatogenesis

### 4.1 SCs maintain the SSCs pools homeostasis

The SSCs niche consists of GCs, SCs, interstitial cells, PMCs, and growth factors from the vascular network (Kanatsu-Shinohara et al., 2007; Oatley and Brinster, 2012). A stable niche facilitates the self-renewal of SSCs and maintains the stem cell pool (Ibtisham et al., 2020). SSCs are located on the basement membrane of the seminiferous tubule, in close contact with SCs (Oatley and Brinster, 2012; Guo et al., 2020). In the presence of SCs, SSCs resume proliferation and undergo several mitotic divisions to form spermatocytes, which give rise to haploid spermatids by meiosis (Zhang et al., 2012). Studies have shown that SCs are activated to produce GDNF in response to FSH, it is a key factor in SSCs self-renewal (Garcia et al., 2017). Ablation of GDNF or its receptors Ret and Gfra1 in SSCs leads to the loss of stem cells and their progeny (Naughton et al., 2006). However, excessive GDNF inhibits differentiation, leading to more undifferentiated SSCs (Hofmann, 2008; Zhang et al., 2012). Furthermore, NOTCH signaling in SCs regulates GDNF expression through HES1 and HEY1 transcription factors, and undifferentiated GCs expressing the NOTCH ligand JAG1 participate in GDNF modulation within the stem cell niche via negative feedback regulation (Garcia et al., 2017). SCs stabilize the SSCs niche by secreting leukemia inhibitory factor (LIF) and chemokines CXCL12 (Oatley et al., 2011; Oatley and Brinster, 2012; Yang et al., 2021). SCs also secrete fibroblast growth factor (FGF), platelet-derived growth factor (PDGF), and Wnt5a, which contribute to SSCs self-renewal (Yeh et al., 2011; Hasegawa and Saga, 2014; Ren et al., 2020). These factors are regulated by FSH signaling, which activates the FSH receptor (FSHR) on SCs and targets functional and transcription factors via the cAMP/PKA pathway (Lim and Hwang, 1995; Sharpe et al., 2003; Wang et al., 2022). Interestingly, exogenous increases SCs leads to a corresponding increase in the number of niches (Oatley et al., 2011). However, excessive self-renewal or differentiation of SSCs can disrupt spermatogenesis, ultimately leading to male infertility (Li S et al., 2021).

### 4.2 SCs create a unique metabolic microenvironment for spermatogenesis

One important event in spermatogenesis is the metabolic cooperation between SCs and developing GCs. After birth, the initial entry into the spermatogenic cycle is triggered by a pubertal upsurge in retinoic acid (RA) (Teletin et al., 2019). RA is an intermediate metabolite of vitamin A (Raverdeau et al., 2012). Within SCs, retinol is oxidized to retinal by RDH10 and then to RA by RALDH1a1 (Theodosiou et al., 2010). RDH10 is essential for the biosynthesis of RA and its absence in SCs results in defective spermatogonia differentiation (Sandell et al., 2012; Tong et al., 2013). RA interacts with heterodimers of the RA receptors (RARs) and the retinoid X receptors (RXRs), binding to RA response elements (RAREs) in target genes, and recruiting co-activators or co-repressors to either induce or inhibit transcription (Mark et al., 2015). Studies have shown that RA deficiency gradually reduces the number of GCs in rats, leaving only SCs and undifferentiated spermatogonia in the seminiferous tubules (Li et al., 2019). Besides, RARα is an important RA member expressed in SCs, when lack RARα, sperm release fails (Vernet et al., 2008). The spermatogenic cycle is supported by subsequent RA pulses (Hogarth et al., 2015; Hofmann and McBeath, 2022). These pulses were observed to influence spermiation at stages VIII–IX of the seminiferous epithelium cycle and were linked to the stage-specific localization of mRNAs associated with retinoid storage, RA synthesis, and degradation (van Pelt and de Rooij, 1990; Vernet et al., 2006). This was confirmed by the discovery of the RA-responsive gene *Stra8*, which is primarily expressed at stages VIII–IX of the seminiferous cycle (Endo et al., 2015).

The testis is a highly specialized organ characterized by low and unevenly distributed oxygen levels. In the testis, SCs exhibit a high glycolytic flux to produce the elevated lactate levels necessary for GCs development (Qian et al., 2023). Glucose is absorbed from the extracellular space via glucose transporter proteins, particularly GLUT1 and GLUT3, and then converted to lactate, in this process, the lactate dehydrogenase system plays a key role by reversibly catalyzing the conversion of pyruvate to lactate (Custódio et al., 2021). Lactate is then transported across the plasma membrane by monocarboxylate transporters (MCT) to the GCs as an energy source (Bernardino et al., 2019). Studies have shown that during spermatogenesis, increased expression of mitochondrial respiratory-related genes in differentiated GCs leads to pyruvate and lactate replacing glucose as the fuel for mitochondrial respiration in spermatogonia and spermatocytes (Mita and Hall, 1982; Nakamura et al., 1984; Ren et al., 2022). Furthermore, intratesticular injection of lactate significantly improved spermatogenesis in the testes of adult cryptorchid rats (Courtens and Plöen, 1999). In this metabolic environment, SCs are co-regulated by both external and internal factors, such as insulin, sex hormones, cytokines, and AMP-activated kinases (Boussouar and Benahmed, 2004). Research suggests that a lack of insulin decreases the expression of LDHA, which in turn reduces the conversion of pyruvate to lactate and MCT4 (Oliveira et al., 2012). These findings align with another study, which demonstrated that insulin and FSH increase lactate production in ISCs, suggesting a positive correlation between insulin levels and lactate production (Oonk et al., 1985).

### 4.3 The blood-testis barrier (BTB) established by SCs is involved in spermatogenesis

The BTB comprises multiple protein complexes between adjacent SCs, including tight junctions (TJ), ectoplasmic specialization (ES, specific adhesion junctions in the testis), gap junctions (GJ) and desmosome junctions (DJ) (Mao et al., 2020). TJ forms barriers dividing the seminiferous epithelium into basal and apical compartments, preventing the diffusion of soluble substances and macromolecules into the apical compartment and limiting the translocation of proteins and lipids between these compartments (Mruk and Cheng, 2015). SCs express occludin and claudin-11, the major regulators of TJ (McCabe et al., 2016). The basal compartment contains SSCs, spermatogonia, and preleptotene spermatocytes (Wanjari and Gopalakrishnan, 2024). Preleptotene spermatocytes enter the apical compartment through the transient opening of the BTB junction complex, where they undergo two meiotic divisions to form spermatocytes, eventually becoming elongated spermatozoa, which enter the lumen of the seminiferous tubules (Cheng and Mruk, 2012). To prevent the self-antigens of spermatocytes and spermatozoa in the apical compartment from triggering an immune response, the BTB isolates many GCs specific antigens, acting as an immune barrier for spermatogenesis (Luaces et al., 2023). ES is mainly composed of cadherin-catenin complexes and can be categorized into two types based on their location: one is basal ES between the SCs (Qian et al., 2014). The other is the apical ES between SCs and spermatids, they interact with the acrosomal region of the spermatid and promote rapid elongation and maturation by mechanically grasping the head of the spermatids (Mruk and Cheng, 2004; Berruti and Paiardi, 2014; Qian et al., 2014). SCs participate in spermatogenesis by providing nutrients (sugars, amino acids) and secreting cytokines (Chojnacka et al., 2016; Luca et al., 2018b). GJ-mediated intercellular substance transport realizes this function in SCs, and GJ can selectively release substances according to those required by GCs at different times (Haverfield et al., 2014; Ni et al., 2019), this regulation of bioactive substance concentration provides a suitable microenvironment for spermatogenesis (O’Donnell et al., 2011). In addition, GJ expresses abundant connexin43 (Pointis et al., 2010), which interacts with Plakophilin2, β-catenin, N-cadherin, and c-Src to mediate signaling during the reconstruction of the seminiferous epithelium in spermatogenesis and regulate the migration of preleptotene spermatocyte (Carette et al., 2010; Gerber et al., 2016).

The BTB is a highly dynamic ultrastructure that undergoes cyclic reorganization in response to various factors, facilitating the completion of meiosis in leptotene spermatocytes (Mruk and Cheng, 2015; Liu et al., 2024). It has been shown that TGF-β and TNF-α regulate BTB reorganization by activating the MAPK signaling pathway, promoting protein endocytosis and ectosome degradation, which interfere with TJ barrier function (Xia et al., 2009; Liu J et al., 2018). Besides, spermatocytes induce SCs to produce and highly IL-1 during stage VIII of the spermatogenic epithelial cycle, regulating the BTB reorganization and spermatozoa release (Sarkar et al., 2008). Androgens promote not only the completion of meiosis and development of spermatocytes (Christin-Maitre and Young, 2022) but also the production of TJ proteins, which maintain the SCs’ barrier function and BTB integrity (McCabe et al., 2012; Kabbesh et al., 2022). In the testis, where androgen concentration fluctuates markedly, the BTB isolates seminiferous tubules from the interstitial tissue, allowing SCs to express androgen-binding protein (ABP) (Walker, 2010; Chen T et al., 2022; Washburn and Dufour, 2023). Androgen binds to ABP, alleviating fluctuations and enabling stable release (Ma et al., 2015). Androgens regulate GCs adhesion and sperm release through the activation of a non-classical testosterone pathway, binding to DNA regulatory elements via androgen receptors (AR) on SCs (Holdcraft and Braun, 2004; Cheng et al., 2007; Deng et al., 2017). Transferrin, located on SCs, transfers iron ions from the blood to cell membranes and transmit them to GCs at different stages, promoting the development, differentiation, and maturation of GCs (Yefimova et al., 2008). Additionally, the BTB confers polarity to the spermatogenic epithelium and guides GCs migration, ensuring proper directional movement of spermatogenic cells (Liu Y et al., 2018). In summary, the BTB established by SCs contributes to spermatogenesis. when damaged, it leads to abnormalities in the testicular microenvironment, including disorganization and loss of polarity of SCs and GCs, disrupting normal spermatogenesis in the testis.

### 4.4 Sperm production and output are directed by SCs

Normal spermatogenesis depends on specific signals from the hypothalamic-pituitary-gonadal axis (HPGA). The hypothalamus produces of gonadotropin-releasing hormone (GnRH), which stimulates the anterior pituitary to synthesize and secrete FSH and LH through activation of its receptor (GnRHR) (Lenzi et al., 2009; Smith and Walker, 2014). However, GCs do not express FSH receptors (FSHR) and must signal through SCs to produce cytokines and metabolites that promote GCs maturation (Meachem et al., 2005; Wu et al., 2015). Specifically, binding of FSH to FSHR induces GTP-binding protein in SCs to recruit other molecules, stimulate Gα_s_ or inhibit Gα_i_, and regulate the cAMP synthesis, subsequently, cAMP activates the downstream transcription factor CREB, maintaining the supportive role of SCs in spermatogenesis (Scobey et al., 2001). LH acts on receptors (LHR) on LCs to promote testosterone synthesis by inducing proliferation, differentiation and maturation of LCs (Smith and Walker, 2014). Testosterone induces spermatogonia to complete meiosis via the AR on SCs, ensuring that GCs acquire the ability to divide (De Gendt et al., 2004; Larose et al., 2020). Testosterone deficiency alters the expression of proteins involved in RNA processing, modification, apoptosis, oxidative stress, and meiosis thereby impeding spermatogenesis (Stanton et al., 2012). Damage to FSH or AR on SCs leads to GCs apoptosis and reduced spermatogenesis efficiency (Ruwanpura et al., 2010; Willems et al., 2010; De Gendt et al., 2014). One study demonstrated that LHR knockout mice lacked testosterone production and exhibited impaired spermatogenesis (Lei et al., 2001), whereas exogenous testosterone administration restored spermatogenesis (Oduwole et al., 2014). It is suggested that SCs play a critical role in transducing of FSH and androgen signaling to coordinate spermatogenesis.

During spermatogenesis, SCs continuously alter their function, cytoskeleton, specialized junctions, gene and protein expression to provide nutrients and physical support to GCs at various stages (Dunleavy et al., 2019). As sperm mature, they need to detach from SCs and enter the seminiferous tubules, eventually being transported to the epididymis for further processing (O’Donnell, 2014). During sperm release, SCs also act as “quality inspectors” by screening and phagocytosing abnormal spermatozoa (O’Donnell et al., 2011). This process dependent on the binding of the scavenger receptor class B type I (SR-B1) in SCs to the membrane phospholipid phosphatidylserine on the target cell (Nakagawa et al., 2005; Zohar-Fux et al., 2022). Phosphatidylserine is normally confined to the inner leaflet of the bilayer membrane, but is transferred to the outer leaflet and exposesd on the cell surface during apoptosis (Park and Kim, 2019). In the process of GCs deformation, a large amount of waste is produced, and SCs recycle this waste for their own energy production (Regueira et al., 2018). Sperm are highly sensitive to microenvironmental changes, and disruptions in SC signaling pathways or drastic hormonal changes can lead to sperm release failure (Kumar et al., 2018). The GCs undergo spermatogenesis to form species-specific elongated spermatids, which are eventually released from the seminiferous epithelium (Du et al., 2021). In the mouse testis, a complete spermatogenic cycle occurs along the seminiferous tubules, while in humans, multiple cycles are intertwined (Johnston et al., 2008; O’Donnell et al., 2022a), meaning that human SCs undergo a more complex process during spermatogenesis.

## 5 SCs determine the fate of LCs

### 5.1 SCs promote LCs differentiation and maturation

At least two types of LCs are present in mammalian embryonic and postnatal testis: FLCs and ALCs (Heinrich and DeFalco, 2020). During early embryonic development, pre-SCs promote the formation of fetal PLCs (Rotgers et al., 2018), once FLCs are appointed, their dependence on FSCs diminishes (Fouchécourt et al., 2016; Mäkelä and Hobbs, 2019). Cytokines secreted by FSCs are involved in the proliferation and survival of FLCs, including KIT ligand (Tsikolia et al., 2009), Desert Hedgehog (DHH) (Yao et al., 2002), and PDGF-A (Brennan et al., 2003). Studies have shown that knocking down DHH in mice reduces the number of FLCs (Yao et al., 2002), while activation of DHH induces the expression of SMO and the downstream transcription factors GLI-1, promoting the proliferation of FLCs (Barsoum et al., 2013). FLCs express PDGFRα, which promotes their differentiation in response to PDGF-A secreted by FSCs (Basciani et al., 2002; Brennan et al., 2003). In contrast, the absence of PDGF-A leads to developmental defects in ALCs but does not affect the number of FLCs (Gnessi et al., 2000). Male gonadal differentiation is facilitated by the interaction of FLCs and FSCs (Svingen and Koopman, 2013; Shima, 2019). Although both FLCs and ALCs are capable of producing androgens (Val et al., 2003), testosterone during the fetal period requires conversion by the 17β-HSD enzyme in FSCs, as FLCs only synthesize androstenedione (O’Shaughnessy et al., 2002). Androgens produced by FLCs and FSCs contribute to the development of the epididymis, seminal vesicles, and vas deferens (O’Donnell et al., 2022b). During the fetal period, genital tissues produce 5α-reductase, which converts testosterone produced by FSCs to dihydrotestosterone, inducing the development of the male urethra, prostate, and penis (Kim et al., 2002; Markouli and Michala, 2023).

After birth, ALCs gradually replace FLCs and begin synthesizing and secreting androgens at puberty (Shima, 2019). The genealogical relationship between FLCs and ALCs has not been fully elucidated. Some scholars believe that ALCs originate from stem cells and are independent of FLCs (Habert, 1993; Lo et al., 2004; Ge et al., 2006; Griswold and Behringer, 2009). Others suggest that a subset of the ALCs arises from a subpopulation of de-differentiated FLCs that remain after birth (Kilcoyne et al., 2014; Kaftanovskaya et al., 2015; Shima et al., 2015; Shima et al., 2018), which is regulated by WT1 in SCs (Wen et al., 2014). WT1 is highly expressed in SCs and induces LCs proliferation and differentiation by regulating DHH in SCs, promoting DHH binding to the Ptch1 receptor on LCs (Chen et al., 2014; Min et al., 2022). WT1 knockout in mice leads to reduced numbers of ALCs, downregulation of steroidogenic enzyme expression, and decreased testosterone levels (Chen et al., 2014). Additionally, Rebourcet’s study found that the number of SCs during development predicts the number of LCs in the adult testis (Rebourcet et al., 2017). Complete ablation of SCs in neonates restricts the differentiation and development of the LCs population (Rebourcet et al., 2014a).

### 5.2 SCs contribute to LCs steroid hormone synthesis

At the onset of spermatogenesis, SCs play a crucial role in providing continuous support to LCs, ensuring their optimal functionality. Studies have shown that the removal of ASCs leads to the apoptosis of ALCs and a significant reduction in their cell number (Rebourcet et al., 2014b). This suggests a dependency between the number of SCs and LCs. SCs secrete various cytokines, such as LIF, PDGF, IGF1, FGF2, TGF, Kit ligand, and Notch ligand, which regulate LCs development and steroidogenesis (Hu et al., 2010; Martin, 2016; Chen M et al., 2017). Among them, IGF1, FGF2 and TGF increased the expression of StAR, promoting the transfer of cholesterol from the outer to the inner mitochondrial membrane to form pregnenolone (Manna et al., 2006). Additionally, SCs can convert testosterone to estrogen via aromatase, which negatively regulates steroidogenesis (Zhang Z. et al., 2022). In co-cultured studies, a reciprocal relationship between SCs and LCs was observed, suggesting that SCs might influence LCs function. In the co-culture system, LCs exhibit a more developed smooth endoplasmic reticulum and higher testosterone content (Saez et al., 1989), while medium collected from SCs increased LCs steroid hormone synthesis (Khan and Rai, 2005). In addition, reducing estrogen levels in SCs promotes testosterone synthesis in co-cultured LCs (Deng et al., 2018). LCs can also influence the metabolic activity of SCs by secreting cytokines (Khan and Rai, 2004). SCs release numerous exosomes, which can cross the BTB to act on LCs functions, providing new evidence for exosomal communication between SCs and LCs. Ma et al. demonstrated that exosomes secreted by SCs promote the survival of LCs by activating the CCL20-AKT pathway (Ma et al., 2022). Exposure to PFOS significantly increases the expression level of miR-9-3p in both SCs and their exosomes, and this miRNA can be transferred to LCs, where it targets and suppresses StAR expression, thereby inhibiting the testosterone synthesis and secretion (Huang et al., 2022). Furthermore, Liang found that miR-145-5p can be transported to LCs via SCs-derived exosomes and exert its function by targeting the *SF-1* gene, which in turn downregulated the expression of steroidogenesis-related genes, ultimately inhibited testosterone production (Liang et al., 2021).

In summary, SCs play a crucial role in regulating the development and function of both FLCs and ALCs. The interactions between these 2 cell types are essential for the spermatogenesis, and their crosstalk may underlie various male reproductive dysfunctions. Further research is needed to elucidate the specific mechanisms of these interactions.

## 6 Effects of SCs on other somatic cells in the testis

### 6.1 Influence of SCs on PMCs

PMCs are the least studied cells in the testis, and their origin remains controversial. Most researchers believe that PMCs and LCs originate from a common progenitor, a mesenchymal stem cell (Ademi et al., 2022; Aksel et al., 2023; Robinson et al., 2023; Tao et al., 2023), This was also confirmed by Guo useing single-cell transcriptome sequencing, who found that in human testis, LCs and PMCs share a common progenitor, and these progenitor cells co-express LCs and PMCs markers (Guo et al., 2020). SCs are essential for the proliferation and differentiation of PMCs. It has been shown that DHH secreted by SCs promotes the proliferation and differentiation of PMCs, and knocked out of the *DHH* gene in SCs results in impaired PMCs during development in the testis (Pierucci-Alves et al., 2001). DHH signaling confer polarity to PMCs in organoid culture studies and promotes reorganization of spermatogenic tubules *in vitro* (Min et al., 2022). SCs also produce WT1, and early reports indicated that specific knockdown of WT1 on SCs using Amh-Cre mice disrupts fetal testicular cords formation (Gao et al., 2006). Wen and colleagues discovered that WT1 regulates the development of FLCs and interstitial progenitor cell lineages through Notch signaling and plays a role in PMCs development, and that the absence of WT1 in SCs leads to reduced differentiation of PMCs and impedes fetal testis compartmentalization (Wen et al., 2016). During testicular cord assembly, PMCs secrete collagen I, collagen IV, and fibronectin, while SCs produce and deposit collagen IV and small amounts of laminin, forming the basal lamina of the seminiferous tubules, which is important for maintaining the ecological niche stability of SSCs after birth (Creemers et al., 2002; Kanatsu-Shinohara et al., 2003; Zhou et al., 2019). The stable number of neonatal SCs facilitates the maintenance of PMCs polarity. When SCs are ablated, PMCs depolarization accelerates, leading to disorganized seminiferous tubules morphology (Rebourcet et al., 2014a; Tao et al., 2023). In the adult testis, SCs ablation does not reduce the number of PMCs but diminishes their activity, which subsequently induces the loss of ALCs (Rebourcet et al., 2014b). It is evident that SCs are crucial for PMCs differentiation and contribute to the maintenance of normal PMCs function in adulthood.

### 6.2 SCs impact on TMs

The testis is a unique immune microenvironment where dendritic cells, mast cells, lymphocytes, and TMs play important roles (Pérez et al., 2013; Bhushan et al., 2015; Bhushan et al., 2020). In the testis, immune responses are generally suppressed, which protects GCs from the immune system and sustaining fertility (Fijak et al., 2011; Li et al., 2012). SCs secrete various immunomodulatory factors that are essential for the function of interstitial immune cells (Luca et al., 2018a). However, SCs derived immunomodulators require androgens support, and when AR are absent in SCs, TJ in SCs are damaged, leading to changes in interstitial immune cells and loss of immune privilege (Meng et al., 2011). TMs constitute a significant portion of the testicular immune microenvironment and are key contributors to its immune function (Bhushan et al., 2020). TMs are present in early undifferentiated fetal gonads (DeFalco et al., 2014; Wang et al., 2021) and are recruited by FSCs to promote organ-specific developmental functions during a narrow time window of embryonic development (Gu et al., 2023). Activin A, a member of the TGF-β superfamily, is primarily secreted by SCs and exerts both pro-inflammatory and anti-inflammatory effects (Hedger and de Krester, 2013; Wijayarathna and de Kretser, 2016), promoting the production of various inflammatory mediators by TMs (de Kretser et al., 2012; Morianos et al., 2019). Activin A also plays a crucial role in regulating macrophage development and function (Hedger and de Kretser, 2013) and helps maintain TMs numbers and testicular immune privilege (Biniwale et al., 2022). It has been long believed that SCs protect GCs from immune attacks by forming the BTB (Kaur et al., 2014; França et al., 2016). However, recent studies suggest that SCs can produce GCs-specific proteins and release them into the interstitial space, potentially promoting peripheral immune tolerance (Tung et al., 2017; O’Donnell et al., 2021). These findings reveal the complex mechanisms by which SCs maintain testicular immune privilege.

Previous research has focused primarily on the interactions between SCs and GCs. However, the specific mechanisms and pathways by which SCs regulate other somatic cells in the testis remain unclear and warrant further investigation. SCs secrete various specific mRNAs and proteins that affect only neighboring somatic and GCs but may also regulate overall male reproductive health. Therefore, an in-depth study of SC-specific gene expression will help elucidate the molecular mechanisms by which SCs modulate the function of somatic cells within the testicular microenvironment.

## 7 Dysfunction of SCs affects male reproduction

SCs are the “custodians” of the spermatogenic microenvironment, the integrity of their structure and function directly determines the efficiency of spermatogenesis and male fertility. The principal drivers affecting SC function fall into three categories: genetic factors, environmental exposures, and lifestyle/dietary patterns. These factors can disrupt BTB, perturb the HPG axis and immune homeostasis, trigger oxidative stress and mitochondrial damage, and remodel metabolic and paracrine signaling, thereby weakening nutritional and protective support for GCs and ultimately leading to impaired spermatogenesis.

### 7.1 Key genes regulation

In most mammals (including humans and mice), early gonadal development is non-dimorphic, generating a primordial, bipotential gonad. Its fate is determined by expression of the sex-determining gene SRY on the Y chromosome (Chen R et al., 2022). SRY cooperates with NR5A1/SF1 to bind the testicular enhancer core (TESCO) sequence, rapidly initiating SOX9 expression and thereby establishing the SC lineage (Sekido and Lovell-Badge, 2008; Gonen et al., 2017). Once activated, SOX9 forms a positive feedback loop through prostaglandin D2 (PGD2) and FGF9, and directly inhibits the RSPO1/WNT4/β-catenin pathway that drives ovarian differentiation, ensuring testis cord formation and AMH expression (Rotgers et al., 2018; Chen and Gao, 2022). During this process, FGF9/FGFR2 signaling is critical for promoting proliferation of precursor cells and for inhibiting WNT4 to stabilize the male developmental trajectory (Bagheri-Fam et al., 2017). Studies also show that loss of *RSPO1* can cause abnormal expansion of androgen-producing cells (Rotgers et al., 2018). The upstream regulator WT1 functions as a hub with multiple roles, it guides somatic cells of the genital ridge toward differentiation into SCs or FLCs during early development; after sex determination, it maintains cell polarity and BTB function by activating non-canonical Wnt pathways (Wnt-PCP and Wnt/Ca2+), while continuously inhibiting β-catenin signaling. Experiments confirm that absence of *Wt1* during the fetal or prepubertal period severely disrupts testicular architecture and LCs development, resulting in male infertility (Rotgers et al., 2018; Liang et al., 2019). Notably, WT1 exerts time-dependent bidirectional control over SF1 expression (enhancing it before sex determination but antagonizing it in SCs thereafter), highlighting WT1’s central role in maintaining lineage stability and the testicular microenvironment (Wilhelm and Englert, 2002; Chen et al., 2012).

In the late differentiation phase and after birth, DMRT1 becomes essential for preserving SCs identity. From E12.5 onward, DMRT1 consolidates male fate by continuously repressing ovarian genes such as *Wnt4* and *Foxl2*, while upregulating *Ptgds*, *Fgfr2*, and *Gdnf* (Matson et al., 2011; Xu et al., 2020). Postnatal loss of DMRT1 leads to transdifferentiation of SCs into granulosa-like cells and can even trigger reversal of gonadal fate (Matson et al., 2011). Furthermore, absence of Raptor, a core component of the mTORC1 pathway, induces dedifferentiation of ASCs, suggesting that nutrient sensing and epigenetic programs jointly establish a metabolic gate that secures mature cellular homeostasis (Xiong et al., 2018). At the hormone regulation level, NR5A1/SF1 directly activates the AMH promoter, and its specific deletion in SCs also downregulates AR expression and delays cellular maturation (Kato et al., 2012; Anamthathmakula et al., 2019), providing a molecular explanation for phenotypes observed in certain pathogenic NR5A1/SF1 variants. X-linked loss-of-function mutations in *AR* are the predominant cause of androgen insensitivity syndrome (AIS), a prototypical SC-related disorder/difference of sex development (DSD). In complete AIS, AR variants are detectable in more than 95% of cases, underscoring the indispensability of this pathway for terminal SCs maturation (Delli Paoli et al., 2023; Karseladze et al., 2024). Importantly, the molecular underpinnings of 46,XY DSD are highly heterogeneous. More than 60 genes have been implicated, and approximately 60% of cases can be attributed to mutations in *AR*, *SRD5A2*, or *NR5A1*/*SF1* (Delot and Vilain, 2021; Zheng et al., 2023). A recent study further identified *DHX37*, *MYRF*, and *PPP2R3C* as 46,XY DSD-associated genes. *DHX37* encodes a DEAD-box RNA helicase; hotspot mutations within its RecA-like domains (e.g., p.R308Q, p.R674W) are strongly associated with gonadal dysgenesis, cryptorchidism, and micropenis, suggesting that ribosome biogenesis, cell-cycle control, and the NF-κB and Wnt signaling pathways may collectively mediate the impact of DHX37 on the SCs lineage (McElreavey et al., 2020; Zhang et al., 2022; Zhang et al., 2025).

### 7.2 Etiologies of sertoli cell-only syndrome

Sertoli cell-only syndrome (SCOS) is the most common and severe subtype of nonobstructive azoospermia (NOA), with a detection rate of 26.3%–69.6% among NOA patients, thereby posing a serious threat to male reproductive health (Chen T et al., 2022). The etiology of SCOS is complex and involves genetic, hormonal, and environmental factors, including Y-chromosome microdeletions, chromosomal abnormalities, cryptorchidism, varicocele, radiotherapy, and harmful occupational exposures (Ghanami Gashti et al., 2021; Hamoda et al., 2025). Studies indicate that karyotypic abnormalities (e.g., Klinefelter syndrome) and microdeletions in the Y-chromosome azoospermia factor (AZF) regions are key markers for SCOS subtyping. The AZF loci are located on the long arm of the Y chromosome (Yq11). There are five fragile sites on Yq11, and their disruption can lead to recurrent deletions of large DNA segments ranging from 0.8 Mb to 7.7 Mb (Wang et al., 2023b; Krausz et al., 2024). Histologically, seminiferous tubules in SCOS contain only SCs, with a complete absence of GCs, and are often accompanied by tubular atrophy and basement membrane thickening (Ghanami Gashti et al., 2021; Wang et al., 2023a). In a minority of cases, focal “residual spermatogenesis” may be present, offering the possibility of biological fatherhood via testicular sperm extraction (TESE) combined with intracytoplasmic sperm injection (ICSI), although this also carries genetic risks for the offspring (Ghanami Gashti et al., 2021; Krausz et al., 2024). Patients with SCOS typically exhibit elevated FSH with largely normal LH and testosterone levels, suggesting compensatory pituitary feedback for spermatogenic failure and relatively preserved LC function (Ghanami Gashti et al., 2021; Wang et al., 2023a). SCOS can be classified as primary or secondary: the former originates from defects in the directed migration or differentiation of PGCs during embryogenesis and most commonly presents as complete SCOS (cSCOS). The latter relates to abnormalities in the maintenance, self-renewal, or differentiation of SSCs after birth, or to dysfunction of the somatic niche, typically presenting early as incomplete SCOS (iSCOS) and potentially progressing over time to cSCOS. The evolution of iSCOS into cSCOS has been demonstrated in multiple genetically engineered models (Wang et al., 2023b), indicating that “intrinsic SSC defects” and “niche failure” are two key mechanisms that converge on a common terminal phenotype. In addition to the classic AZF deletion, aberrant expression of genes such as *NANOS1*, *SYCP3*, *TEX11*, and *HSF* has been implicated in SCOS (Chen T et al., 2022; Wang et al., 2023a). Transcriptomic analyses further show that downregulated genes in SCOS are enriched in cell-cycle and reproduction-related pathways, whereas upregulated genes are associated with inflammation and immune responses. Protein–protein interaction network analysis identifies hub genes centered on *CCNB1*, *AURKA*, and *CDC20*, these all belong to suppressed cell-cycle modules, suggesting that spermatogenic arrest is accompanied by marked cell-cycle dysregulation (Chen R et al., 2022). Additionally, immune cell infiltration is increased in SCOS samples, and CD56^bright^ NK cells are significantly associated with multiple hub genes, indicating that “cell-cycle arrest” and “activation of the immune microenvironment” together constitute the dual molecular underpinnings of SCOS. In addition, noncoding RNAs participate in niche imbalance and the regulation of SSC fate by modulating signaling pathways such as Notch, EGFR, and LIF/IL-6, thereby providing new insights for a comprehensive elucidation of SCOS pathogenesis (Hofmann and McBeath, 2022; Wang et al., 2023b).

### 7.3 Sertoli cell tumor

Sertoli cell tumor (SCT) is the second most common testicular sex cord–stromal tumor in males and accounts for approximately 1% of all testicular neoplasms. Based on histopathological features, SCTs are categorized into two types: not otherwise specified (NOS) and large cell calcifying SCT (LCCSCT) (Patrikidou et al., 2023). LCCSCT occurs in prepubertal and postpubertal patients, and some of them are associated with cancer-predisposition syndrome and germline variations (Al-Obaidy et al., 2022). SCT may arise from system-level imbalances of SCs in the multilayered development and homeostatic regulatory network and involves three interrelated pathological modules. First, the non-classical Wnt/PCP pathway regulated by WT1 maintains cell polarity and lineage stability, whereas the classical Wnt/β-catenin pathway is physiologically restrained to preserve the resting state and the BTB. When this regulatory axis is perturbed, it drives aberrant proliferation and dedifferentiation of SCs (Wang et al., 2013; Lobo et al., 2023). Evidence indicates that miR-202-3p targets LRP6 and cyclin D1 within the Wnt/β-catenin cascade, thereby restraining cell-cycle progression (Yang et al., 2019), suggesting that “enhancement of Wnt signaling” together with “cell-cycle propulsion” constitutes a key pro-proliferative circuit in SCs whose molecular features closely align with tumorigenesis. In addition to abnormalities in the Wnt pathway, loss of the homeostatic functions of developmental transcription factors further accelerates malignant transformation; for example, SF1/NR5A1 regulates the survival and ordered proliferation of embryonic SCs through the MDM2/TP53 signaling pathway (Anamthathmakula et al., 2019), and imbalance of this pathway may attenuate TP53-dependent checkpoint control, permitting expansion of aberrant clones. Finally, epigenetic disequilibrium amplifies these processes. Pervasive histone acetylation abnormalities have been observed in testes with SCOS (Ghanami Gashti et al., 2021), indicating systemic alterations in the SC microenvironment and chromatin openness; this provides an epigenetic basis for sustained activation of pro-proliferative transcriptional programs, including the Wnt–cyclin D axis. Notably, some SCOS pathological specimens show loss of epithelial characteristics in SCs accompanied by disruption of seminiferous tubule architecture (Peirouvi et al., 2021). This deterioration of the tissue microenvironment confers spatial growth advantages and immune-evasion conditions to abnormal clones, providing a plausible explanation for the clinically observed low-grade malignant tendency and risk of local recurrence in SCT.

### 7.4 Systemic aging

SCs are the most age-sensitive cell type in the testis; compared to young individuals, the number of age-related differentially expressed genes increases in elderly testes (Huang et al., 2023). Systemic factors associated with aging, along with changes in the local microenvironment, reduce the number of SCs and impair their function, further affecting spermatogenesis (Rebourcet et al., 2017; Xiao et al., 2024). At the ultrastructural level, aging leads to changes in SCs, including abnormal nuclear morphology, periodic loss of organelles, relaxation of the endoplasmic reticulum, and enlarged lysosomes, resulting in a decline in their function (Santiago et al., 2019).

Specifically, aging leads to lipid metabolism disorders in SCs. Studies have shown that genes involved in sterol biosynthesis and transport (such as *Msmo1*, *Sqle*, *Tspo*, *Hmgcr*, *Elovl5*, *Fads1*, and *Dhcr24*) are upregulated in aged SCs (Nie et al., 2022), indicating the accumulation of lipid inclusions within SCs (Paniagua et al., 1987; Santiago et al., 2019). Sulfogalactosylglycerolipid (SGG) is essential for spermatogenesis (Tanphaichitr et al., 2018), and arylsulfatase A (ARSA) produced by SCs can degrade SGG in apoptotic GCs and their remnants (Kongmanas et al., 2021). When SCs fail to degrade SGG promptly, lipid droplet accumulation occurs. However, whether reduced ARSA activity in senescent SCs leads to SGG accumulation, accompanied by higher ROS levels, thereby affecting spermatogenesis, requires further investigation. Moreover, SCs secrete various signaling molecules, and their secretory capacity gradually declines with aging. For example, GDNF secreted by SCs regulates the self-renewal of SSCs (Parekh et al., 2019), but its expression levels in SCs decrease with advancing age (Ryu et al., 2006). This may be one of the key reasons for the reduced number of SSCs in aged individuals. Additionally, periodic RA pulses serve as key regulators of spermatogenesis (Endo et al., 2017; Saracino et al., 2020). The RA receptor signaling pathway is significantly decreased in aged SCs (Huang et al., 2023). This suggests that aged SCs have a reduced capacity to guide spermatocyte differentiation, resulting in slower GCs entry into meiosis. scRNA-seq analysis further revealed that key transcription regulators such as WT1, GATA4, and AR exhibited significantly reduced expression levels in aged SCs (Huang et al., 2023). This will affect the expression levels of FYN, a gene closely associated with GCs’ survival and differentiation (Luo et al., 2012; Yang et al., 2022). In addition to secretory functions, SCs also have prominent phagocytic functions (Tysoe, 2024). During spermatogenesis, SCs are responsible for clearing metabolic waste and residual cytoplasm generated throughout the process (Yefimova et al., 2018; Tysoe, 2024). However, in aged animals, the seminiferous tubules harbor substantial accumulations of residual bodies and arrested round spermatids that have not been cleared. This buildup may disrupt the microenvironmental homeostasis of the seminiferous tubules and impede spermatogenesis (Dong et al., 2016). Additionally, increased expression of inflammation-related genes (such as *Il1a*, *Il6st*, *Ifi27*, *Ccl2*, and *Mif*) was observed in aged SCs (Nie et al., 2022). Given the nutritional and supportive role of SCs in the GCs lineage, these altered transcriptional levels indicate a decline in overall metabolic function and heightened inflammation in aged SCs, thereby impairing their capacity to support GCs development.

### 7.5 Endocrine disrupting chemicals

Endocrine disrupting chemicals (EDCs) are pollutants widely present in the environment, originating largely from the pharmaceutical, cosmetics, and packaging industries. These substances can enter the human body, interfere with normal endocrine activity, and adversely affect male reproductive health (Feijo et al., 2025). In the male reproductive system, EDCs impair the function of SCs by inhibiting proliferation, disrupting the cytoskeleton and BTB integrity, and interfering with adhesion junctions between SCs and GCs, ultimately leading to testicular dysfunction and infertility (Mitra et al., 2017; Stukenborg et al., 2021; Adegoke et al., 2023). Common EDCs that may adversely affect male reproductive health include cadmium, phthalates, bisphenol A (BPA), perfluorinated compounds, pesticides, among others (Gao et al., 2025).

As industrial development continues, heavy metals are increasingly released into the environment, either intentionally or unintentionally, harming human and animal health through inhalation or consumption of contaminated water sources. Cadmium (Cd), a prevalent environmental pollutant from industrial processes and cigarette smoking, has been shown to accumulate in the body, particularly in the testes, despite a daily intake as low as 1.06 μg/kg body weight (Wan et al., 2013). This accumulation can disrupt various bodily functions. SCs are a primary target of Cd, pregnant rats on GD12 received low-dose intraperitoneal injections of Cd, which led to decreased expression *DHH* and *FSHR* genes in SCs, although it did not significantly affect SCs number, it may have impacted the development and function of other testicular cells (Li et al., 2018). Cd exposure in lactating rats led to vacuolization of SCs and loss of spermatogenic epithelial cells in adult rats (Bekheet, 2011). Additionally, Cd induces excessive autophagy in SCs, leading to apoptosis and an increase in reactive ROS (Chen et al., 2024). SCs support spermatogenesis by forming the BTB (Luaces et al., 2023). However, in rats, Cd disrupts BTB structure by upregulating TGF-β3, which activates p38MAPK signaling (Lui et al., 2003; Wong et al., 2004). Focal adhesion kinase (FAK), a non-receptor protein tyrosine kinase, regulates the TJ proteins (such as occludin and ZO-1) in the BTB, and Cd downregulates FAK expression, damaging the BTB and further inhibiting spermatogenesis (Venditti et al., 2021; Li et al., 2024).

BPA, a prototypical environmental estrogen, is a significant EDC that negatively affects male reproductive health. Most human exposure to BPA occurs through household items, medical devices, and food packaging made from polycarbonate plastics (Corpuz-Hilsabeck and Culty, 2023). BPA can induce metabolic disorders and abnormal spermatogenesis through mechanisms including mimicking estrogenic activity, interfering with DNA methylation, and altering enzyme activities (Barbagallo et al., 2020; Deng et al., 2024). Numerous studies indicate that the reproductive toxicity of BPA primarily arises from impairment of SCs function (Corpuz-Hilsabeck and Culty, 2023). During fetal development, the effects of BPA are complex. One study reported that intrauterine BPA exposure downregulates the expression of AMH, a marker of male fetal SCs, and reduces testosterone levels, suggesting impaired function of both SCs and LCs (Lv et al., 2019). However, an organ culture study observed that BPA exposure increased numbers and markers of FSCs in mice, while GCs and FLCs were significantly reduced, the authors therefore hypothesized that BPA may disrupt the normal proportions and cellular communication among various testicular cell types, and that this imbalance in cellular niche serving as a key mechanism of reproductive dysfunction (Park et al., 2021). Rossi et al. examined primary SCs isolated from 7-day-old postnatal mice and found that exposure to 0.5 μM BPA enhanced endocannabinoid (eCB) signaling, a lipid-based signaling system that acts on cannabinoid receptors and is known to regulate spermatogenesis and sperm fertilization capacity. Because FSH regulates eCB signaling in ISCs, the authors propose that BPA may disrupt FSH signaling (Rossi et al., 2020). In a TM4 cell model, BPA showed a biphasic effect: micromolar levels of BPA inhibited cell proliferation, whereas nanomolar levels stimulated energy metabolism, evidenced by elevated ATP levels and increased mitochondrial activity (Ge et al., 2014). Additionally, BPA induces apoptosis in adolescent rat SCs (PND 18–22) via activating the Fas/FasL and p38/JNK pathways (Qi et al., 2014). Co-exposure to BPA and phthalates has been reported to synergistically disrupt Sertoli–germ cell tight and gap junctions, markedly reducing AR and junctional protein expression and thereby compromising BTB integrity (de Freitas et al., 2016).

### 7.6 Lifestyle/dietary patterns

In recent years, rapid socio-economic development, combined with unhealthy eating habits and poor lifestyles, has led to a steady increase in global obesity rates (Chooi et al., 2019). Obesity is a major risk factor for non-communicable diseases and is strongly associated with diabetes, fatty liver disease, cardiovascular disease, and cancer (Lauby-Secretan et al., 2016). Obesity has been shown to cause reproductive dysfunction in men, and excess body fat affecting sperm production and quality, making men more susceptible to oligozoospermia and azoospermia (Ma et al., 2019). Previous studies have found that obesity impairs spermatogenesis by disrupting the BTB (Zhang et al., 2014). Recent studies suggest that male obesity cause hormonal imbalances in the HPGA, leading to secondary hypogonadism (Fernandez et al., 2019). Specifically, white adipose tissue produces high levels of cytochrome P450 aromatase, which converts androgens to estrogens, and estrogens then act on the hypothalamus through negative feedback, inhibiting the production and secretion of FSH and LH, thereby impairing testosterone synthesis and spermatogenesis (Barbagallo et al., 2021). The inflammasome NLRP3 is a sensor of cellular damage, once activated, it recruits ASCs to form a caspase-1 activation platform, inducing interleukin secretion and creating an inflammatory microenvironment (Karki and Kanneganti, 2019). Researchers found that decreased AMPKα expression and increased NADPH oxidase activity activated NLRP3 expression in the testes of obese mice, further exacerbating obesity induced SCs dysfunction and male infertility (Mu et al., 2022). Leptin, produced by adipocytes, regulates the body’s energy balance, however, plasma leptin concentrations are high in most obese individuals and are proportional to systemic adiposity. Evidence suggests that leptin promotes the phosphorylation of IRS family members in the hypothalamus, and that phosphorylation of IRS inhibitory sites can impair interactions between FSH and IGF1 in SCs, potentially affecting spermatogenesis (Duan et al., 2004). The impairment of spermatogenesis caused by obesity is regulated by multiple factors, and the specific mechanisms remain to be further investigated.

## 8 Conclusion and outlook

Male infertility is a major public health issue affecting approximately 10% of couples of reproductive age worldwide, with 50% of cases attributable to male factors (Agarwal et al., 2021). In recent years, male reproductive health in industrialized countries has significantly deteriorated, as evidenced by reduced sperm quality (Lv et al., 2021), lower serum testosterone levels (Travison et al., 2009), and an increased incidence of testicular cancer (Skakkebæk et al., 2022). Consequently, elucidating the mechanisms that regulate testicular function is especially important. In this context, SCs play a central role. They participate in testicular development and gonadal differentiation, by secreting various cytokines, act together with GCs, LCs, and PMCs to form a complex signaling network that maintains homeostasis of the testicular microenvironment. Existing studies indicate that functional impairment of SCs is closely associated with endocrine disruptors, oxidative stress, inflammation, and other environmental factors. However, most current research on SCs relies on two-dimensional culture models that struggle to accurately recapitulate the complex *in vivo* environment. Moreover, although multi-omics technologies (including proteomics, epigenomics, transcriptomics, and metabolomics) can identify key molecules in SCs, major challenges remain in systematically integrating multi-omics datasets, building reliable biomarker networks, and validating them effectively *in vivo*. To address these gaps, future research should focus on developing organoids and three-dimensional culture systems to construct testicular microenvironment models with greater physiological relevance. By integrating multi-omics data and fostering interdisciplinary collaboration, a systematic dissection of SC regulatory networks under physiological and pathological conditions may provide stronger theoretical foundations and new therapeutic strategies for the mechanistic study and clinical intervention of male infertility.

## References

[B1] Adegoke E. O. Rahman M. S. Amjad S. Pang W. K. Ryu D. Y. Park Y. J. (2023). Environmentally relevant doses of endocrine disrupting chemicals affect Male fertility by interfering with sertoli cell glucose metabolism in mice. Chemosphere 337, 139277. 10.1016/j.chemosphere.2023.139277 37364641

[B2] Ademi H. Djari C. Mayère C. Neirijnck Y. Sararols P. Rands C. M. (2022). Deciphering the origins and fates of steroidogenic lineages in the mouse testis. Cell Rep. 39 (11), 110935. 10.1016/j.celrep.2022.110935 35705036

[B3] Agarwal A. Baskaran S. Parekh N. Cho C.-L. Henkel R. Vij S. (2021). Male infertility. Lancet 397 (10271), 319–333. 10.1016/S0140-6736(20)32667-2 33308486

[B4] Aksel S. Cao M. Derpinghaus A. Baskin L. S. Cunha G. R. (2023). Ontogeny of mouse Sertoli, Leydig and peritubular myoid cells from embryonic day 10 to adulthood. Differentiation 129, 96–108. 10.1016/j.diff.2022.02.006 35317954

[B5] Al-Obaidy K. I. Idrees M. T. Abdulfatah E. Kunju L. P. Wu A. Ulbright T. M. (2022). Large cell calcifying sertoli cell tumor: a clinicopathologic Study of 18 cases with comprehensive review of the literature and reappraisal of Prognostic features. Am. J. Surg. Pathol. 46 (5), 688–700. 10.1097/PAS.0000000000001849 34913878

[B6] Albrecht K. H. Eicher E. M. (2001). Evidence that Sry is expressed in pre-Sertoli cells and Sertoli and granulosa cells have a common precursor. Dev. Biol. 240 (1), 92–107. 10.1006/dbio.2001.0438 11784049

[B7] Anamthathmakula P. Miryala C. S. J. Moreci R. S. Kyathanahalli C. Hassan S. S. Condon J. C. (2019). Steroidogenic factor 1 (Nr5a1) is required for Sertoli cell survival post sex determination. Sci. Rep. 9 (1), 4452. 10.1038/s41598-019-41051-1 30872705 PMC6418149

[B8] Archambeault D. R. Yao H. H. (2010). Activin A, a product of fetal Leydig cells, is a unique paracrine regulator of Sertoli cell proliferation and fetal testis cord expansion. Proc. Natl. Acad. Sci. U. S. A. 107 (23), 10526–10531. 10.1073/pnas.1000318107 20498064 PMC2890803

[B9] Archambeault D. R. Yao H. H. (2014). Loss of smad4 in Sertoli and Leydig cells leads to testicular dysgenesis and hemorrhagic tumor formation in mice. Biol. Reprod. 90 (3), 62. 10.1095/biolreprod.113.111393 24501173 PMC4076392

[B10] Arora H. Qureshi R. Khodamoradi K. Seetharam D. Parmar M. Van Booven D. J. (2022). Leptin secreted from testicular microenvironment modulates hedgehog signaling to augment the endogenous function of Leydig cells. Cell Death and Dis. 13 (3), 208. 10.1038/s41419-022-04658-3 35246515 PMC8897450

[B11] Bagheri-Fam S. Argentaro A. Svingen T. Combes A. N. Sinclair A. H. Koopman P. (2011). Defective survival of proliferating Sertoli cells and androgen receptor function in a mouse model of the ATR-X syndrome. Hum. Mol. Genet. 20 (17), 3535. 10.1093/hmg/ddr267 21427128

[B12] Bagheri-Fam S. Bird A. D. Zhao L. Ryan J. M. Yong M. Wilhelm D. (2017). Testis determination requires a specific FGFR2 Isoform to repress FOXL2. Endocrinology 158 (11), 3832–3843. 10.1210/en.2017-00674 28938467 PMC5695826

[B13] Bale T. L. Epperson C. N. (2015). Sex differences and stress across the lifespan. Nat. Neurosci. 18 (10), 1413–1420. 10.1038/nn.4112 26404716 PMC4620712

[B14] Barbagallo F. Condorelli R. A. Mongioi L. M. Cannarella R. Aversa A. Calogero A. E. (2020). Effects of bisphenols on testicular steroidogenesis. Front. Endocrinol. 11, 373. 10.3389/fendo.2020.00373 32714277 PMC7344146

[B15] Barbagallo F. Condorelli R. A. Mongioi L. M. Cannarella R. Cimino L. Magagnini M. C. (2021). Molecular mechanisms underlying the relationship between obesity and Male infertility. Metabolites 11 (12), 840. 10.3390/metabo11120840 34940598 PMC8706114

[B16] Barbara P. D. Méjean C. Moniot B. Malclès M. H. Berta P. Boizet-Bonhoure B. (2001). Steroidogenic factor-1 contributes to the cyclic-adenosine monophosphate down-regulation of human SRY gene expression. Biol. Reprod. 64 (3), 775–783. 10.1095/biolreprod64.3.775 11207191

[B17] Barsoum I. B. Bingham N. C. Parker K. L. Jorgensen J. S. Yao H. H. C. (2009). Activation of the Hedgehog pathway in the mouse fetal ovary leads to ectopic appearance of fetal Leydig cells and female pseudohermaphroditism. Dev. Biol. 329 (1), 96–103. 10.1016/j.ydbio.2009.02.025 19268447 PMC2673990

[B18] Barsoum I. B. Kaur J. Ge R. S. Cooke P. S. Yao H. H. C. (2013). Dynamic changes in fetal Leydig cell populations influence adult Leydig cell populations in mice. Faseb J. 27 (7), 2657–2666. 10.1096/fj.12-225060 23568777 PMC3688755

[B19] Basciani S. Mariani S. Arizzi M. Ulisse S. Rucci N. Jannini E. A. (2002). Expression of platelet-derived growth factor-A (PDGF-A), PDGF-B, and PDGF receptor-alpha and -beta during human testicular development and disease. J. Clin. Endocrinol. Metab. 87 (5), 2310–2319. 10.1210/jcem.87.5.8476 11994382

[B20] Bekheet S. H. M. (2011). Comparative effects of repeated administration of cadmium chloride during pregnancy and lactation and selenium protection against cadmium toxicity on some organs in immature rats' offsprings. Biol. Trace Elem. Res. 144 (1-3), 1008–1023. 10.1007/s12011-011-9084-z 21614561

[B21] Bernardino R. L. D'Souza W. N. Rato L. Rothstein J. L. Dias T. R. Chui D. (2019). Knockout of MCT1 results in total absence of spermatozoa, sex hormones dysregulation, and morphological alterations in the testicular tissue. Cell Tissue Res. 378 (2), 333–339. 10.1007/s00441-019-03028-4 31073907

[B22] Berruti G. Paiardi C. (2014). The dynamic of the apical ectoplasmic specialization between spermatids and Sertoli cells: the case of the small GTPase Rap1. Biomed. Res. Int. 2014, 635979. 10.1155/2014/635979 24719879 PMC3955676

[B23] Bhushan S. Meinhardt A. (2017). The macrophages in testis function. J. Reprod. Immunol. 119, 107–112. 10.1016/j.jri.2016.06.008 27422223

[B24] Bhushan S. Tchatalbachev S. Lu Y. N. Fröhlich S. Fijak M. Vijayan V. (2015). Differential activation of inflammatory pathways in testicular macrophages provides a rationale for their subdued inflammatory capacity. J. Immunol. 194 (11), 5455–5464. 10.4049/jimmunol.1401132 25917085

[B25] Bhushan S. Theas M. S. Guazzone V. A. Jacobo P. Wang M. Fijak M. (2020). Immune cell subtypes and their function in the testis. Front. Immunol. 11, 583304. 10.3389/fimmu.2020.583304 33101311 PMC7554629

[B26] Biniwale S. Wijayarathna R. Pleuger C. Bhushan S. Loveland K. L. Meinhardt A. (2022). Regulation of macrophage number and gene transcript levels by activin A and its binding protein, follistatin, in the testes of adult mice. J. Reprod. Immunol. 151, 103618. 10.1016/j.jri.2022.103618 35378491

[B27] Birk O. S. Casiano D. E. Wassif C. A. Cogliati T. Zhao L. Zhao Y. (2000). The LIM homeobox gene Lhx9 is essential for mouse gonad formation. Nature 403 (6772), 909–913. 10.1038/35002622 10706291

[B28] Boussouar F. Benahmed M. (2004). Lactate and energy metabolism in male germ cells. Trends Endocrinol. Metab. 15 (7), 345–350. 10.1016/j.tem.2004.07.003 15350607

[B29] Brennan J. Capel B. (2004). One tissue, two fates: molecular genetic events that underlie testis *versus* ovary development. Nat. Rev. Genet. 5 (7), 509–521. 10.1038/nrg1381 15211353

[B30] Brennan J. Tilmann C. Capel B. (2003). Pdgfr-alpha mediates testis cord organization and fetal Leydig cell development in the XY gonad. Genes and Dev. 17 (6), 800–810. 10.1101/gad.1052503 12651897 PMC196020

[B31] Bullejos M. Koopman P. (2001). Spatially dynamic expression of Sry in mouse genital ridges. Dev. Dyn. 221 (2), 201–205. 10.1002/dvdy.1134 11376487

[B32] Bunce C. Barske L. Zhang G. R. Capel B. (2023). Biased precursor ingression underlies the center-to-pole pattern of male sex determination in mouse. Development 150 (5), dev201060. 10.1242/dev.201060 36912416 PMC10112898

[B33] Cannarella R. Arato I. Condorelli R. A. Luca G. Barbagallo F. Alamo A. (2019). The IGF1 receptor is involved in follicle-stimulating hormone signaling in porcine Neonatal Sertoli cells. J. Clin. Med. 8 (5), 577. 10.3390/jcm8050577 31035547 PMC6571966

[B34] Carette D. Weider K. Gilleron J. Giese S. Dompierre J. Bergmann M. (2010). Major involvement of connexin 43 in seminiferous epithelial junction dynamics and male fertility. Dev. Biol. 346 (1), 54–67. 10.1016/j.ydbio.2010.07.014 20655897

[B35] Carré G. A. Greenfield A. (2016). The gonadal supporting cell lineage and Mammalian sex determination: the differentiation of Sertoli and granulosa cells. Results Probl. Cell Differ. 58, 47–66. 10.1007/978-3-319-31973-5_3 27300175

[B36] Chan Y. F. Tang F. S. O W. (2008). Adrenomedullin in the rat testis. II: its production, actions on inhibin secretion, regulation by follicle-stimulating hormone, and its interaction with endothelin 1 in the Sertoli cell. Biol. Reprod. 78 (4), 780–785. 10.1095/biolreprod.107.060863 18094364

[B37] Chauvigné F. Verdura S. Mazón M. J. Duncan N. Zanuy S. Gómez A. (2012). Follicle-Stimulating hormone and luteinizing hormone mediate the androgenic pathway in Leydig cells of an evolutionary advanced teleost. Biol. Reprod. 87 (2), 35. 10.1095/biolreprod.112.100784 22649073

[B38] Chen M. Gao F. (2022). The regulation of gonadal somatic cell differentiation in humans. Genomics Proteomics Bioinforma. 20 (2), 219–222. 10.1016/j.gpb.2022.04.003 35504504 PMC9684145

[B39] Chen S. R. Liu Y. X. (2016). Testis cord maintenance in Mouse embryos: genes and signaling. Biol. Reprod. 94 (2), 42. 10.1095/biolreprod.115.137117 26792939

[B40] Chen G. C. Yang L. Q. Begum S. Xu L. (2010). GPR56 is essential for Testis development and Male fertility in mice. Dev. Dyn. 239 (12), 3358–3367. 10.1002/dvdy.22468 20981830 PMC2991479

[B41] Chen H. Palmer J. S. Thiagarajan R. D. Dinger M. E. Lesieur E. Chiu H. (2012). Identification of novel markers of mouse fetal ovary development. PLoS One 7 (7), e41683. 10.1371/journal.pone.0041683 22844512 PMC3406020

[B42] Chen M. Wang X. Wang Y. Zhang L. Xu B. Lv L. (2014). Wt1 is involved in leydig cell steroid hormone biosynthesis by regulating paracrine factor expression in mice. Biol. Reprod. 90 (4), 71. 10.1095/biolreprod.113.114702 24571983

[B43] Chen L. Y. Willis W. D. Eddy E. M. (2016). Targeting the Gdnf Gene in peritubular myoid cells disrupts undifferentiated spermatogonial cell development. Proc. Natl. Acad. Sci. U. S. A. 113 (7), 1829–1834. 10.1073/pnas.1517994113 26831079 PMC4763785

[B44] Chen H. Murray E. Sinha A. Laumas A. Li J. Lesman D. (2021). Dissecting mammalian spermatogenesis using spatial transcriptomics. Cell Rep. 37 (5), 109915. 10.1016/j.celrep.2021.109915 34731600 PMC8606188

[B45] Chen N. Wan X. Y. Cheng S. Tang G. J. Xia D. Xu Y. L. (2024). Defective autophagic flux aggravates cadmium-induced Sertoli cell apoptosis. Ecotoxicol. Environ. Saf. 273, 116095. 10.1016/j.ecoenv.2024.116095 38367604

[B46] Chen H. Wang Y. Y. Ge R. S. Zirkin B. R. (2017). Leydig cell stem cells: identification, proliferation and differentiation. Mol. Cell. Endocrinol. 445 (C), 65–73. 10.1016/j.mce.2016.10.010 27743991 PMC5346484

[B47] Chen M M. Zhang L. Cui X. Lin X. Li Y. Wang Y. (2017). Wt1 directs the lineage specification of sertoli and granulosa cells by repressing Sf1 expression. Development 144 (1), 44–53. 10.1242/dev.144105 27888191

[B48] Chen R. Wang F. Chen Y. Han D. (2022). Immune homeostasis and disorder in the testis - roles of Sertoli cells. J. Reprod. Immunol. 152, 103625. 10.1016/j.jri.2022.103625 35580404

[B49] Chen T. Wang Y. Tian L. Guo X. Xia J. Wang Z. (2022). Aberrant gene expression profiling in men with Sertoli cell-only syndrome. Front. Immunol. 13, 821010. 10.3389/fimmu.2022.821010 35833143 PMC9273009

[B50] Cheng C. Y. Mruk D. D. (2012). The blood-testis barrier and its implications for Male contraception. Pharmacol. Rev. 64 (1), 16–64. 10.1124/pr.110.002790 22039149 PMC3250082

[B51] Cheng J. Watkins S. C. Walker W. H. (2007). Testosterone activates mitogen-activated protein kinase *via* Src kinase and the epidermal growth factor receptor in sertoli cells. Endocrinology 148 (5), 2066–2074. 10.1210/en.2006-1465 17272394

[B52] Chojnacka K. Zarzycka M. Mruk D. D. (2016). Biology of the sertoli cell in the fetal, pubertal, and adult Mammalian testis. Results Probl. Cell Differ. 58, 225–251. 10.1007/978-3-319-31973-5_9 27300181

[B53] Chooi Y. C. Ding C. Magkos F. (2019). The epidemiology of obesity. Metabolism-Clinical Exp. 92, 6–10. 10.1016/j.metabol.2018.09.005 30253139

[B54] Christin-Maitre S. Young J. (2022). Androgens and spermatogenesis. Ann. D. Endocrinol. 83 (3), 155–158. 10.1016/j.ando.2022.04.010 35489414

[B55] Chui K. Trivedi A. Cheng C. Y. Cherbavaz D. B. Dazin P. F. Huynh A. L. (2011). Characterization and functionality of proliferative human Sertoli cells. Cell Transpl. 20 (5), 619–635. 10.3727/096368910x536563 21054948 PMC4096632

[B56] Combes A. N. Wilhelm D. Davidson T. Dejana E. Harley V. Sinclair A. (2009). Endothelial cell migration directs testis cord formation. Dev. Biol. 326 (1), 112–120. 10.1016/j.ydbio.2008.10.040 19041858

[B57] Cool J. DeFalco T. Capel B. (2012). Testis formation in the fetal mouse: dynamic and complex *de novo* tubulogenesis. Wiley Interdiscip. Reviews-Developmental Biol. 1 (6), 847–859. 10.1002/wdev.62 23799626

[B58] Corpuz-Hilsabeck M. Culty M. (2023). Impact of endocrine disrupting chemicals and pharmaceuticals on Sertoli cell development and functions. Front. Endocrinol. (Lausanne) 14, 1095894. 10.3389/fendo.2023.1095894 36793282 PMC9922725

[B59] Courtens J. L. Plöen L. (1999). Improvement of spermatogenesis in adult cryptorchid rat testis by intratesticular infusion of lactate. Biol. Reprod. 61 (1), 154–161. 10.1095/biolreprod61.1.154 10377044

[B60] Creemers L. B. Meng X. den Ouden K. van Pelt A. M. M. Izadyar F. Santoro M. (2002). Transplantation of germ cells from glial cell line-derived neurotrophic factor-overexpressing mice to host testes depleted of endogenous spermatogenesis by fractionated irradiation. Biol. Reprod. 66(6): 1579–1584. 10.1095/biolreprod66.6.1579 12021034

[B61] Custódio T. F. Paulsen P. A. Frain K. M. Pedersen B. P. (2021). Structural comparison of GLUT1 to GLUT3 reveal transport regulation mechanism in sugar porter family. Life Sci. Alliance 4 (4), e202000858. 10.26508/lsa.202000858 33536238 PMC7898563

[B62] de Freitas A. Ribeiro M. A. Pinho C. F. Peixoto A. R. Domeniconi R. F. Scarano W. R. (2016). Regulatory and junctional proteins of the blood-testis barrier in human Sertoli cells are modified by monobutyl phthalate (MBP) and bisphenol A (BPA) exposure. Toxicol. Vitro 34, 1–7. 10.1016/j.tiv.2016.02.017 26922907

[B63] De Gendt K. Swinnen J. V. Saunders P. T. K. Schoonjans L. Dewerchin M. Devos A. (2004). A Sertoli cell-selective knockout of the androgen receptor causes spermatogenic arrest in meiosis. Proc. Natl. Acad. Sci. U. S. A. 101 (5), 1327–1332. 10.1073/pnas.0308114100 14745012 PMC337052

[B64] De Gendt K. Verhoeven G. Amieux P. S. Wilkinson M. F. (2014). Research resource: Genome-Wide identification of AR-Regulated genes translated in Sertoli cells *in vivo* using the RiboTag approach. Mol. Endocrinol. 28 (4), 575–591. 10.1210/me.2013-1391 24606126 PMC3968405

[B65] de Kretser D. M. O'Hehir R. E. Hardy C. L. Hedger M. P. (2012). The roles of activin A and its binding protein, follistatin, in inflammation and tissue repair. Mol. Cell. Endocrinol. 359 (1-2), 101–106. 10.1016/j.mce.2011.10.009 22037168

[B66] De Rooij D. G. Russell L. D. (2000). All you wanted to know about spermatogonia but were afraid to ask. J. Androl. 21 (6), 776–798. 10.1002/j.1939-4640.2000.tb03408.x 11105904

[B67] DeFalco T. Bhattacharya I. Williams A. V. Sams D. M. Capel B. (2014). Yolk-sac-derived macrophages regulate fetal testis vascularization and morphogenesis. Proc. Natl. Acad. Sci. U. S. A. 111 (23), E2384–E2393. 10.1073/pnas.1400057111 24912173 PMC4060703

[B68] DeFalco T. Potter S. J. Williams A. V. Waller B. Kan M. J. Capel B. (2015). Macrophages contribute to the spermatogonial niche in the adult testis. Cell Rep. 12 (7), 1107–1119. 10.1016/j.celrep.2015.07.015 26257171 PMC4545310

[B69] del Campo A. B. Kyte J. A. Carretero J. Zinchencko S. Mendez R. Gonzalez-Aseguinolaza G. (2014). Immune escape of cancer cells with beta2-microglobulin loss over the course of metastatic melanoma. Int. J. Cancer 134 (1), 102–113. 10.1002/ijc.28338 23784959

[B70] Del Valle I. Buonocore F. Duncan A. J. Lin L. Barenco M. Parnaik R. (2017). A genomic atlas of human adrenal and gonad development. Wellcome Open Res. 2, 25. 10.12688/wellcomeopenres.11253.2 28459107 PMC5407452

[B71] Delli Paoli E. Di Chiano S. Paoli D. Lenzi A. Lombardo F. Pallotti F. (2023). Androgen insensitivity syndrome: a review. J. Endocrinol. Invest 46 (11), 2237–2245. 10.1007/s40618-023-02127-y 37300628

[B72] Delot E. C. Vilain E. (2021). Towards improved genetic diagnosis of human differences of sex development. Nat. Rev. Genet. 22 (9), 588–602. 10.1038/s41576-021-00365-5 34083777 PMC10598994

[B73] Deng Q. Wu Y. Zhang Z. Wang Y. Li M. H. Liang H. (2017). Androgen receptor localizes to plasma membrane by binding to Caveolin-1 in mouse Sertoli cells. Int. J. Endocrinol. 2017, 3985916. 10.1155/2017/3985916 28642789 PMC5470003

[B74] Deng S. L. Wang Z. P. Jin C. Kang X. L. Batool A. Zhang Y. (2018). Melatonin promotes sheep Leydig cell testosterone secretion in a co-culture with Sertoli cells. Theriogenology 106, 170–177. 10.1016/j.theriogenology.2017.10.025 29073541

[B75] Deng X. Liang S. Tang Y. Li Y. Xu R. Luo L. (2024). Adverse effects of bisphenol A and its analogues on male fertility: an epigenetic perspective. Environ. Pollut. 345, 123393. 10.1016/j.envpol.2024.123393 38266695

[B76] Dong Y. S. Hou W. G. Li Y. Liu D. B. Hao G. Z. Zhang H. F. (2016). Unexpected requirement for a binding partner of the syntaxin family in phagocytosis by murine testicular Sertoli cells. Cell Death Differ. 23 (5), 787–800. 10.1038/cdd.2015.139 26494466 PMC4832098

[B77] Du L. Chen W. Cheng Z. X. Wu S. He J. Han L. (2021). Novel gene regulation in normal and abnormal spermatogenesis. Cells 10 (3), 666. 10.3390/cells10030666 33802813 PMC8002376

[B78] Duan C. J. Li M. H. Rui L. Y. (2004). SH2-B promotes insulin receptor substrate 1 (IRS1)- and IRS2-mediated activation of the phosphatidylinositol 3-kinase pathway in response to leptin. J. Biol. Chem. 279 (42), 43684–43691. 10.1074/jbc.M408495200 15316008 PMC3874232

[B79] Dunleavy J. E. M. O'Bryan M. K. Stanton P. G. O'Donnell L. (2019). The cytoskeleton in spermatogenesis. Reproduction 157 (2), R53–R72. 10.1530/Rep-18-0457 30576284

[B80] Dym M. Kokkinaki M. He Z. (2009). Spermatogonial stem cells: mouse and human comparisons. Birth Defects Res. C Embryo Today 87 (1), 27–34. 10.1002/bdrc.20141 19306345

[B81] Endo T. Romer K. A. Anderson E. L. Baltus A. E. de Rooij D. G. Page D. C. (2015). Periodic retinoic acid-STRA8 signaling intersects with periodic germ-cell competencies to regulate spermatogenesis. Proc. Natl. Acad. Sci. U. S. A. 112 (18), E2347–E2356. 10.1073/pnas.1505683112 25902548 PMC4426408

[B82] Endo T. Freinkman E. de Rooij D. G. Page D. C. (2017). Periodic production of retinoic acid by meiotic and somatic cells coordinates four transitions in mouse spermatogenesis. Proc. Natl. Acad. Sci. U. S. A. 114 (47), E10132–E10141. 10.1073/pnas.1710837114 29109271 PMC5703301

[B83] Fadlalla K. (2019). 99 temporal and spatial characterization of interleukin-6 protein in the caprine testis. J. Anim. Sci. 97, 83. 10.1093/jas/skz258.171

[B84] Fang Y. W. Su Y. F. Xu J. Hu Z. Y. Zhao K. Liu C. Y. (2021). Varicocele-Mediated Male infertility: from the perspective of testicular immunity and inflammation. Front. Immunol. 12, 729539. 10.3389/fimmu.2021.729539 34531872 PMC8438154

[B85] Fang X. Nie L. Putluri S. Ni N. Bartholin L. Li Q. (2023). Sertoli cell-specific activation of transforming growth factor beta receptor 1 leads to testicular granulosa cell tumor Formation. Cells 12 (23), 2717. 10.3390/cells12232717 38067144 PMC10706251

[B86] Fayomi A. P. Orwig K. E. (2018). Spermatogonial stem cells and spermatogenesis in mice, monkeys and men. Stem Cell Res. 29, 207–214. 10.1016/j.scr.2018.04.009 29730571 PMC6010318

[B87] Feijo M. Carvalho T. M. A. Fonseca L. R. S. Vaz C. V. Pereira B. J. Cavaco J. E. B. (2025). Endocrine-disrupting chemicals as prostate carcinogens. Nat. Rev. Urol. 22 (9), 609–631. 10.1038/s41585-025-01031-9 40379948

[B88] Fernandez D. Bertoldi M. V. Gomez L. Morales A. Callegari E. Lopez L. A. (2008). Identification and characterization of Myosin from rat testicular peritubular myoid cells. Biol. Reprod. 79 (6), 1210–1218. 10.1095/biolreprod.107.066472 18716291 PMC2586039

[B89] Fernandez C. J. Chacko E. C. Pappachan J. M. (2019). Male obesity-related secondary hypogonadism - pathophysiology, clinical implications and management. Eur. Endocrinol. 15 (2), 83–90. 10.17925/ee.2019.15.2.83 31616498 PMC6785957

[B90] Fijak M. Bhushan S. Meinhardt A. (2011). Immunoprivileged sites: the testis. Methods Mol. Biol. 677, 459–470. 10.1007/978-1-60761-869-0_29 20941627

[B91] Fleck D. Kenzler L. Mundt N. Strauch M. Uesaka N. Moosmann R. (2021). ATP activation of peritubular cells drives testicular sperm transport. Elife 10, e62885. 10.7554/eLife.62885 33502316 PMC7840184

[B92] Fouchécourt S. Livera G. Messiaen S. Fumel B. Parent A. S. Marine J. C. (2016). Apoptosis of Sertoli cells after conditional ablation of murine double minute 2 (Mdm2) gene is p53-dependent and results in male sterility. Cell Death Differ. 23 (3), 521–530. 10.1038/cdd.2015.120 26470726 PMC5072445

[B93] França L. R. Hess R. A. Dufour J. M. Hofmann M. C. Griswold M. D. (2016). The Sertoli cell: one hundred fifty years of beauty and plasticity. Andrology 4 (2), 189–212. 10.1111/andr.12165 26846984 PMC5461925

[B94] Gao F. Maiti S. Alam N. Zhang Z. Deng J. M. Behringer R. R. (2006). The Wilms tumor gene, Wt1, is required for Sox9 expression and maintenance of tubular architecture in the developing testis. Proc. Natl. Acad. Sci. U. S. A. 103 (32), 11987–11992. 10.1073/pnas.0600994103 16877546 PMC1567685

[B95] Gao S. Fang X. Guo L. (2025). Construction of testicular organoids and their applications in the field of toxicology. Arch. Toxicol. 99 (12), 4737–4755. 10.1007/s00204-025-04177-y 40883615

[B96] Garcia T. X. Parekh P. Gandhi P. Sinha K. Hofmann M. C. (2017). The NOTCH ligand JAG1 regulates GDNF expression in Sertoli cells. Stem Cells Dev. 26 (8), 585–598. 10.1089/scd.2016.0318 28051360 PMC5393414

[B97] Gaytan F. Bellido C. Aguilar E. Vanrooijen N. (1994). Requirement for testicular macrophages in leydig-cell proliferation and differentiation during prepubertal development in rats. J. Reprod. Fertil. 102 (2), 393–399. 10.1530/jrf.0.1020393 7861393

[B98] Ge R. S. Dong Q. A. Sottas C. M. Papadopoulos V. Zirkin B. R. Hardy M. P. (2006). In search of rat stem Leydig cells: identification, isolation, and lineage-specific development. Proc. Natl. Acad. Sci. U. S. A. 103 (8), 2719–2724. 10.1073/pnas.0507692103 16467141 PMC1413776

[B99] Ge L. C. Chen Z. J. Liu H. Zhang K. S. Su Q. Ma X. Y. (2014). Signaling related with biphasic effects of bisphenol A (BPA) on Sertoli cell proliferation: a comparative proteomic analysis. Biochim. Biophys. Acta 1840 (9), 2663–2673. 10.1016/j.bbagen.2014.05.018 24909818

[B100] Gerber J. Heinrich J. Brehm R. (2016). Blood-testis barrier and Sertoli cell function: lessons from SCCx43KO mice. Reproduction 151 (2), R15–R27. 10.1530/Rep-15-0366 26556893

[B101] Ghanami Gashti N. Sadighi Gilani M. A. Abbasi M. (2021). Sertoli cell-only syndrome: etiology and clinical management. J. Assist. Reprod. Genet. 38 (3), 559–572. 10.1007/s10815-021-02063-x 33428073 PMC7910341

[B102] Gnessi L. Basciani S. Mariani S. Arizzi M. Spera G. Wang C. (2000). Leydig cell loss and spermatogenic arrest in platelet-derived growth factor (PDGF)-A-deficient mice. J. Cell Biol. 149 (5), 1019–1026. 10.1083/jcb.149.5.1019 10831606 PMC2174827

[B103] Gonen N. Quinn A. O'Neill H. C. Koopman P. Lovell-Badge R. (2017). Correction: normal levels of Sox9 expression in the developing mouse Testis depend on the TES/TESCO enhancer, but this does not Act alone. PLoS Genet. 13 (2), e1006584. 10.1371/journal.pgen.1006584 28146551 PMC5287446

[B104] Griswold M. D. (2018). 50 years of spermatogenesis: sertoli cells and their interactions with germ cells. Biol. Reprod. 99 (1), 87–100. 10.1093/biolre/ioy027 29462262 PMC7328471

[B105] Griswold S. L. Behringer R. R. (2009). Fetal Leydig cell origin and development. Sex. Dev. 3 (1), 1–15. 10.1159/000200077 19339813 PMC4021856

[B106] Gu X. W. Heinrich A. Li S. Y. DeFalco T. (2023). Testicular macrophages are recruited during a narrow fetal time window and promote organ-specific developmental functions. Nat. Commun. 14 (1), 1439. 10.1038/s41467-023-37199-0 36922518 PMC10017703

[B107] Guo J. Nie X. Giebler M. Mlcochova H. Wang Y. Grow E. J. (2020). The dynamic transcriptional cell Atlas of testis development during human puberty. Cell Stem Cell 26 (2), 262–276 e264. 10.1016/j.stem.2019.12.005 31928944 PMC7298616

[B108] Habert R. (1993). *In vivo* acute testicular testosterone response to injection of luteinizing hormone in the rat fetus. Acta Endocrinol. (Copenh) 128 (3), 268–273. 10.1530/acta.0.1280268 8480478

[B109] Hales D. B. (2002). Testicular macrophage modulation of Leydig cell steroidogenesis. J. Reprod. Immunol. 57 (1-2), 3–18. 10.1016/S0165-0378(02)00020-7 12385830

[B110] Hamoda T. Shah R. Mostafa T. Pinggera G. M. Atmoko W. Rambhatla A. (2025). Global Andrology Forum (GAF) Clinical Guidelines on the management of non-obstructive azoospermia: bridging the gap between controversy and consensus. World J. Mens. Health 43, e26. 10.5534/wjmh.250037 40583014 PMC12798425

[B111] Hancock G. V. Wamaitha S. E. Peretz L. Clark A. T. (2021). Mammalian primordial germ cell specification. Development 148 (6), dev189217. 10.1242/dev.189217 33722957 PMC7990907

[B112] Hanley N. A. Hagan D. M. Clement-Jones M. Ball S. G. Strachan T. Salas-Cortés L. (2000). SRY, SOX9, and DAX1 expression patterns during human sex determination and gonadal development. Mech. Dev. 91 (1-2), 403–407. 10.1016/S0925-4773(99)00307-X 10704874

[B113] Hartmann K. Bennien J. Wapelhorst B. Bakhaus K. Schumacher V. Kliesch S. (2016). Current insights into the sulfatase pathway in human testis and cultured Sertoli cells. Histochem Cell Biol. 146 (6), 737–748. 10.1007/s00418-016-1503-y 27688058

[B114] Hasegawa K. Saga Y. (2014). FGF8-FGFR1 signaling acts as a niche factor for maintaining undifferentiated spermatogonia in the mouse. Biol. Reprod. 91 (6), 145. 10.1095/biolreprod.114.121012 25359900

[B115] Haverfield J. T. Meachem S. J. Nicholls P. K. Rainczuk K. E. Simpson E. R. Stanton P. G. (2014). Differential permeability of the blood-testis barrier during reinitiation of spermatogenesis in adult Male rats. Endocrinology 155 (3), 1131–1144. 10.1210/en.2013-1878 24424039

[B116] Hedger M. P. de Kretser D. M. (2013). The activins and their binding protein, follistatin-diagnostic and therapeutic targets in inflammatory disease and fibrosis. Cytokine and Growth Factor Rev. 24 (3), 285–295. 10.1016/j.cytogfr.2013.03.003 23541927

[B117] Heinrich A. DeFalco T. (2020). Essential roles of interstitial cells in testicular development and function. Andrology 8 (4), 903–914. 10.1111/andr.12703 31444950 PMC7036326

[B118] Hiramatsu R. Matoba S. Kanai-Azuma M. Tsunekawa N. Katoh-Fukui Y. Kurohmaru M. (2009). A critical time window of Sry action in gonadal sex determination in mice. Development 136 (1), 129–138. 10.1242/dev.029587 19036799

[B119] Hofmann M. C. (2008). Gdnf signaling pathways within the mammalian spermatogonial stem cell niche. Mol. Cell. Endocrinol. 288 (1-2), 95–103. 10.1016/j.mce.2008.04.012 18485583 PMC2491722

[B120] Hofmann M. C. McBeath E. (2022). Sertoli cell-germ cell interactions within the niche: paracrine and Juxtacrine molecular communications. Front. Endocrinol. 13, 897062. 10.3389/fendo.2022.897062 35757413 PMC9226676

[B121] Hogarth C. A. Arnold S. Kent T. Mitchell D. Isoherranen N. Griswold M. D. (2015). Processive pulses of retinoic acid Propel asynchronous and continuous murine sperm production. Biol. Reprod. 92 (2), 37. 10.1095/biolreprod.114.126326 25519186 PMC4326729

[B122] Holdcraft R. W. Braun R. E. (2004). Androgen receptor function is required in Sertoli cells for the terminal differentiation of haploid spermatids. Development 131 (2), 459–467. 10.1242/dev.00957 14701682

[B123] Hossain A. Saunders G. F. (2001). The human sex-determining gene SRY is a direct target of WT1. J. Biol. Chem. 276 (20), 16817–16823. 10.1074/jbc.M009056200 11278460

[B124] Hu G. X. Lin H. Chen G. R. Chen B. B. Lian Q. Q. Hardy D. O. (2010). Deletion of the Igf1 gene: suppressive effects on adult Leydig cell development. J. Androl. 31 (4), 379–387. 10.2164/jandrol.109.008680 20203337 PMC4103413

[B125] Huang J. Ren H. Chen A. Li T. Wang H. Jiang L. (2022). Perfluorooctane sulfonate induces suppression of testosterone biosynthesis *via* Sertoli cell-derived exosomal/miR-9-3p downregulating StAR expression in Leydig cells. Environ. Pollut. 301, 118960. 10.1016/j.envpol.2022.118960 35150797

[B126] Huang D. Zuo Y. Zhang C. Sun G. Jing Y. Lei J. (2023). A single-nucleus transcriptomic atlas of primate testicular aging reveals exhaustion of the spermatogonial stem cell reservoir and loss of Sertoli cell homeostasis. Protein Cell 14 (12), 888–907. 10.1093/procel/pwac057 36929025 PMC10691849

[B127] Hume D. A. Halpin D. Charlton H. Gordon S. (1984). The mononuclear phagocyte system of the mouse defined by immunohistochemical localization of antigen F4/80: macrophages of endocrine organs. Proc. Natl. Acad. Sci. U. S. A. 81 (13), 4174–4177. 10.1073/pnas.81.13.4174 6377311 PMC345391

[B128] Hutson J. C. (2006). Physiologic interactions between macrophages and Leydig cells. Exp. Biol. Med. 231 (1), 1–7. 10.1177/153537020623100101 16380639

[B129] Hutson J. M. (2013). Journal of Pediatric Surgery-Sponsored Fred McLoed Lecture. Undescended testis: the underlying mechanisms and the effects on germ cells that cause infertility and cancer. J. Pediatr. Surg. 48 (5), 903–908. 10.1016/j.jpedsurg.2013.02.001 23701757

[B130] Ibtisham F. Awang-Junaidi A. H. Honaramooz A. (2020). The study and manipulation of spermatogonial stem cells using animal models. Cell Tissue Res. 380 (2), 393–414. 10.1007/s00441-020-03212-x 32337615

[B131] Jiménez R. Burgos M. Barrionuevo F. J. (2021). Sex maintenance in mammals. Genes (Basel) 12 (7), 999. 10.3390/genes12070999 34209938 PMC8303465

[B132] Johnston D. S. Wright W. W. DiCandeloro P. Wilson E. Kopf G. S. Jelinsky S. A. (2008). Stage-specific gene expression is a fundamental characteristic of rat spermatogenic cells and Sertoli cells. Proc. Natl. Acad. Sci. U. S. A. 105 (24), 8315–8320. 10.1073/pnas.0709854105 18544648 PMC2448834

[B133] Jones S. L. Styles J. Geyer C. B. (2021). A new translation and reader's guide to the first detailed description of the first wave of spermatogenesis in the mouse. Mol. Reprod. Dev. 88 (7), 473–478. 10.1002/mrd.23519 34096665 PMC8316303

[B134] Kabbesh H. Bulldan A. Konrad L. Scheiner-Bobis G. (2022). The role of ZIP9 and androgen receptor in the establishment of tight junctions between adult rat Sertoli cells. Biology-Basel 11 (5), 668. 10.3390/biology11050668 35625396 PMC9138102

[B135] Kaftanovskaya E. M. Lopez C. Ferguson L. Myhr C. Agoulnik A. I. (2015). Genetic ablation of androgen receptor signaling in fetal Leydig cell lineage affects Leydig cell functions in adult testis. Faseb J. 29 (6), 2327–2337. 10.1096/fj.14-263632 25713029 PMC6137449

[B136] Kanatsu-Shinohara M. Ogonuki N. Inoue K. Miki H. Ogura A. Toyokuni S. (2003). Long-term proliferation in culture and germline transmission of mouse male germline stem cells. Biol. Reprod. 69 (2), 612–616. 10.1095/biolreprod.103.017012 12700182

[B137] Kanatsu-Shinohara M. Inoue K. Ogonuki N. Miki H. Yoshida S. Toyokuni S. (2007). Leukemia inhibitory factor enhances formation of germ cell colonies in neonatal mouse testis culture. Biol. Reprod. 76 (1), 55–62. 10.1095/biolreprod.106.055863 17021343

[B138] Karki R. Kanneganti T. D. (2019). Diverging inflammasome signals in tumorigenesis and potential targeting. Nat. Rev. Cancer 19 (4), 197–214. 10.1038/s41568-019-0123-y 30842595 PMC6953422

[B139] Karl J. Capel B. (1998). Sertoli cells of the mouse testis originate from the coelomic epithelium. Dev. Biol. 203 (2), 323–333. 10.1006/dbio.1998.9068 9808783

[B140] Karseladze A. I. Asaturova A. V. Kiseleva I. A. Badlaeva A. S. Tregubova A. V. Zaretsky A. R. (2024). Androgen insensitivity syndrome with bilateral gonadal sertoli cell lesions, Sertoli-Leydig cell tumor, and paratesticular leiomyoma: a case report and first systematic literature review. J. Clin. Med. 13 (4), 929. 10.3390/jcm13040929 38398243 PMC10889606

[B141] Kato T. Esaki M. Matsuzawa A. Ikeda Y. (2012). NR5A1 is required for functional maturation of Sertoli cells during postnatal development. Reproduction 143 (5), 663–672. 10.1530/REP-11-0365 22419830

[B142] Katoh-Fukui Y. Miyabayashi K. Komatsu T. Owaki A. Baba T. Shima Y. (2012). Cbx2, a polycomb group gene, is required for Sry gene expression in mice. Endocrinology 153 (2), 913–924. 10.1210/en.2011-1055 22186409

[B143] Kaur G. Thompson L. A. Dufour J. M. (2014). Sertoli cells - immunological sentinels of spermatogenesis. Seminars Cell and Dev. Biol. 30, 36–44. 10.1016/j.semcdb.2014.02.011 24603046 PMC4043859

[B144] Kawabe Y. Numabe T. Tanemura K. Hara K. (2022). Characteristics of alpha smooth muscle actin-positive peritubular cells in prepubertal bovine testes. Biochem. Biophys. Res. Commun. 609, 48–53. 10.1016/j.bbrc.2022.03.149 35413539

[B145] Khan U. W. Rai U. (2004). *In vitro* effect of FSH and testosterone on Sertoli cell nursing function in wall lizard Hemidactylus flaviviridis (Rüppell) ruppell. General Comp. Endocrinol. 136 (2), 225–231. 10.1016/j.ygcen.2003.12.015 15028526

[B146] Khan U. W. Rai U. (2005). Endocrine and paracrine control of Leydig cell steroidogenesis and proliferation in the wall lizard: an *in vitro* study. General Comp. Endocrinol. 140 (2), 109–115. 10.1016/j.ygcen.2004.10.009 15613273

[B147] Kilcoyne K. R. Smith L. B. Atanassova N. Macpherson S. McKinnell C. van den Driesche S. (2014). Fetal programming of adult Leydig cell function by androgenic effects on stem/progenitor cells. Proc. Natl. Acad. Sci. U. S. A. 111 (18), E1924–E1932. 10.1073/pnas.1320735111 24753613 PMC4020050

[B148] Kim K. S. Liu W. Cunha G. R. Russell D. W. Huang H. Shapiro E. (2002). Expression of the androgen receptor and 5 alpha-reductase type 2 in the developing human fetal penis and urethra. Cell Tissue Res. 307 (2), 145–153. 10.1007/s004410100464 11845321

[B149] Klattig J. Sierig R. Kruspe D. Makki M. S. Englert C. (2007). WT1-mediated gene regulation in early urogenital ridge development. Sex. Dev. 1 (4), 238–254. 10.1159/000104774 18391535

[B150] Kongmanas K. Saewu A. Kiattiburut W. Baker M. A. Faull K. F. Burger D. (2021). Accumulation of seminolipid in Sertoli cells is associated with increased levels of reactive oxygen species and Male subfertility: studies in aging Arsa null Male mice. Antioxidants (Basel) 10 (6), 912. 10.3390/antiox10060912 34199863 PMC8227610

[B151] Krausz C. Navarro-Costa P. Wilke M. Tuttelmann F. (2024). EAA/EMQN best practice guidelines for molecular diagnosis of Y-chromosomal microdeletions: state of the art 2023. Andrology 12 (3), 487–504. 10.1111/andr.13514 37674303

[B152] Kubota H. Brinster R. L. (2018). Spermatogonial stem cells. Biol. Reprod. 99 (1), 52–74. 10.1093/biolre/ioy077 29617903 PMC6692861

[B153] Kumar A. Raut S. Balasinor N. H. (2018). Endocrine regulation of sperm release. Reprod. Fertil. Dev. 30 (12), 1595–1603. 10.1071/Rd18057 29860969

[B154] Kuroki S. Tachibana M. (2018). Epigenetic regulation of mammalian sex determination. Mol. Cell. Endocrinol. 468, 31–38. 10.1016/j.mce.2017.12.006 29248548

[B155] Larney C. Bailey T. L. Koopman P. (2014). Switching on sex: transcriptional regulation of the testis-determining gene Sry. Development 141 (11), 2195–2205. 10.1242/dev.107052 24866114 PMC4034426

[B156] Larose H. Kent T. Ma Q. Y. Shami A. N. Harerimana N. Li J. Z. (2020). Regulation of meiotic progression by Sertoli-cell androgen signaling. Mol. Biol. Cell 31 (25), 2841–2862. 10.1091/mbc.E20-05-0334 33026960 PMC7851862

[B157] Lauby-Secretan B. Scoccianti C. Loomis D. Grosse Y. Bianchini F. Straif K. (2016). Body fatness and cancer - viewpoint of the IARC Working Group. N. Engl. J. Med. 375 (8), 794–798. 10.1056/NEJMsr1606602 27557308 PMC6754861

[B158] Lee R. Lee W. Y. Park H. J. (2024). Diuron-induced fetal Leydig cell dysfunction in *in vitro* organ cultured fetal testes. Reprod. Toxicol. 123, 108497. 10.1016/j.reprotox.2023.108497 37949197

[B159] Lei Z. M. Mishra S. Zou W. Xu B. Foltz M. Li X. (2001). Targeted disruption of luteinizing hormone/human chorionic gonadotropin receptor gene. Mol. Endocrinol. 15 (1), 184–200. 10.1210/me.15.1.184 11145749

[B160] Lenzi A. Balercia G. Bellastella A. Colao A. Fabbri A. Foresta C. (2009). Epidemiology, diagnosis, and treatment of male hypogonadotropic hypogonadism. J. Endocrinol. Investig. 32 (11), 934–938. 10.1007/Bf03345775 19955846

[B161] Li N. Wang T. Han D. S. (2012). Structural, cellular and molecular aspects of immune privilege in the testis. Front. Immunol. 3, 152. 10.3389/fimmu.2012.00152 22701457 PMC3371599

[B162] Li X. Wang Z. Jiang Z. Guo J. Zhang Y. Li C. (2016). Regulation of seminiferous tubule-associated stem Leydig cells in adult rat testes. Proc. Natl. Acad. Sci. U. S. A. 113 (10), 2666–2671. 10.1073/pnas.1519395113 26929346 PMC4790979

[B163] Li X. J. Liu J. P. Wu S. W. Zheng W. W. Li H. T. Bao S. H. (2018). *In utero* single low-dose exposure of cadmium induces rat fetal Leydig cell dysfunction. Chemosphere 194, 57–66. 10.1016/j.chemosphere.2017.11.159 29197250

[B164] Li X. Long X. Y. Xie Y. J. Zeng X. Chen X. Mo Z. C. (2019). The roles of retinoic acid in the differentiation of spermatogonia and spermatogenic disorders. Clin. Chim. Acta 497, 54–60. 10.1016/j.cca.2019.07.013 31302099

[B165] Li Y. X. Chen Y. G. Wu W. P. Li N. Hua J. L. (2023). MMPs, ADAMs and ADAMTSs are associated with mammalian sperm fate. Theriogenology 200, 147–154. 10.1016/j.theriogenology.2023.02.013 36842259

[B166] Li X.-W. Li S. Yang Y. Talukder M. Xu X.-W. Li C.-X. (2024). The FAK/occludin/ZO-1 complex is critical for cadmium-induced testicular damage by disruption of the integrity of the blood-testis barrier in chickens. J. Hazard. Mater. 470, 134126. 10.1016/j.jhazmat.2024.134126 38554509

[B167] Li Q. Li H. H. Liang J. L. Mei J. X. Cao Z. Zhang L. (2021). Sertoli cell-derived exosomal MicroRNA-486-5p regulates differentiation of spermatogonial stem cell through PTEN in mice. J. Cell. Mol. Med. 25 (8), 3950–3962. 10.1111/jcmm.16347 33608983 PMC8051706

[B168] Li S Y. Gu X. W. Heinrich A. Hurley E. G. Capel B. DeFalco T. (2021). Loss of Mafb and Maf distorts myeloid cell ratios and disrupts fetal mouse testis vascularization and organogenesis†. Biol. Reprod. 105 (4), 958–975. 10.1093/biolre/ioab098 34007995 PMC8511659

[B169] Liang J. Wang N. He J. Du J. Guo Y. Li L. (2019). Induction of Sertoli-like cells from human fibroblasts by NR5A1 and GATA4. Elife 8, e48767. 10.7554/eLife.48767 31710289 PMC6881147

[B170] Liang J. L. Li H. H. Mei J. X. Cao Z. Tang Y. Huang R. F. (2021). Sertoli cell-derived exosome-mediated transfer of miR-145-5p inhibits Leydig cell steroidogenesis by targeting steroidogenic factor 1. Faseb J. 35 (6), e21660. 10.1096/fj.202002589RRRR 34010469 PMC12315942

[B171] Lim K. Hwang B. D. (1995). Follicle-Stimulating-Hormone transiently induces expression of protooncogene C-Myc in primary sertoli-cell cultures of early pubertal and prepubertal rat. Mol. Cell. Endocrinol. 111 (1), 51–56. 10.1016/0303-7207(95)03543-G 7649352

[B172] Liu C. Rodriguez K. Yao H. H. C. (2016). Mapping lineage progression of somatic progenitor cells in the mouse fetal testis. Development 143 (20), 3700–3710. 10.1242/dev.135756 27621062 PMC5087644

[B173] Liu M. R. He Q. Yuan Z. H. Chen N. N. Ren S. Du Q. (2024). HDAC3 promotes Sertoli cell maturation and maintains the blood-testis barrier dynamics. Faseb J. 38 (5), e23526. 10.1096/fj.202301349RR 38430456

[B174] Liu J. Ren L. H. Wei J. L. Zhang J. Zhu Y. P. Li X. Y. (2018). Fine particle matter disrupts the blood-testis barrier by activating TGF-β3/p38 MAPK pathway and decreasing testosterone secretion in rat. Environ. Toxicol. 33 (7), 711–719. 10.1002/tox.22556 29673083

[B175] Liu Y. Hu Y. Wang L. Xu C. (2018). Expression of transcriptional factor EB (TFEB) in differentiating spermatogonia potentially promotes cell migration in mouse seminiferous epithelium. Reprod. Biol. Endocrinol. 16 (1), 105. 10.1186/s12958-018-0427-x 30360758 PMC6202848

[B176] Lo K. C. Lei Z. M. Rao C. V. Beck J. Lamb D. J. (2004). *De novo* testosterone production in luteinizing hormone receptor knockout mice after transplantation of leydig stem cells. Endocrinology 145 (9), 4011–4015. 10.1210/en.2003-1729 15123536

[B177] Lobo J. Acosta A. M. Netto G. J. (2023). Molecular biomarkers with potential clinical application in testicular cancer. Mod. Pathol. 36 (10), 100307. 10.1016/j.modpat.2023.100307 37611872

[B178] Lord T. Oatley J. M. (2017). A revised A_single_ model to explain stem cell dynamics in the mouse male germline. Reproduction 154 (2), R55–R64. 10.1530/Rep-17-0034 28624768 PMC5512591

[B179] Luaces J. P. Toro-Urrego N. Otero-Losada M. Capani F. (2023). What do we know about blood-testis barrier? Current understanding of its structure and physiology. Front. Cell Dev. Biol. 11, 1114769. 10.3389/fcell.2023.1114769 37397257 PMC10307970

[B180] Luca G. Arato I. Sorci G. Cameron D. F. Hansen B. C. Baroni T. (2018a). Sertoli cells for cell transplantation: pre-clinical studies and future perspectives. Andrology 6 (3), 385–395. 10.1111/andr.12484 29600532

[B181] Luca G. Baroni T. Arato I. Hansen B. C. Cameron D. F. Calafiore R. (2018b). Role of Sertoli cell proteins in immunomodulation. Protein Pept. Lett. 25 (5), 440–445. 10.2174/0929866525666180412163151 29651939

[B182] Lucas-Herald A. K. Mitchell R. T. (2022). Testicular sertoli cell hormones in differences in sex development. Front. Endocrinol. 13, 919670. 10.3389/fendo.2022.919670 35909548 PMC9329667

[B183] Lui W. Y. Wong C. H. Mruk D. D. Cheng C. Y. (2003). TGF-beta3 regulates the blood-testis barrier dynamics *via* the p38 mitogen activated protein (MAP) kinase pathway: an *in vivo* study. Endocrinology 144 (4), 1139–1142. 10.1210/en.2002-0211 12639893

[B184] Luo J. Gupta V. Kern B. Tash J. S. Sanchez G. Blanco G. (2012). Role of FYN kinase in spermatogenesis: defects characteristic of Fyn-null sperm in mice. Biol. Reprod. 86 (1), 1–8. 10.1095/biolreprod.111.093864 21918125 PMC3313667

[B185] Luo D. He Z. Yu C. Guan Q. (2022). Role of p38 MAPK signalling in Testis development and Male fertility. Oxid. Med. Cell Longev. 2022, 6891897. 10.1155/2022/6891897 36092154 PMC9453003

[B186] Lv Y. Li L. Fang Y. Chen P. Wu S. Chen X. (2019). In utero exposure to bisphenol A disrupts fetal testis development in rats. Environ. Pollut. 246, 217–224. 10.1016/j.envpol.2018.12.006 30557795

[B187] Lv M. Q. Ge P. Zhang J. Yang Y. Q. Zhou L. Zhou D. X. (2021). Temporal trends in semen concentration and count among 327 373 Chinese healthy men from 1981 to 2019: a systematic review. Hum. Reprod. 36 (7), 1751–1775. 10.1093/humrep/deab124 34046659

[B188] Ma Y. Yang H. Z. Xu L. M. Huang Y. R. Dai H. L. Kang X. N. (2015). Testosterone regulates the autophagic clearance of androgen binding protein in rat Sertoli cells. Sci. Rep. 5, 8894. 10.1038/srep08894 25745956 PMC4352847

[B189] Ma J. X. Wu L. Zhou Y. Zhang H. Xiong C. L. Peng Z. (2019). Association between BMI and semen quality: an observational study of 3966 sperm donors. Hum. Reprod. 34 (1), 155–162. 10.1093/humrep/dey328 30407511 PMC6676949

[B190] Ma Y. Zhou Y. Zou S. S. Sun Y. Chen X. F. (2022). Exosomes released from Sertoli cells contribute to the survival of Leydig cells through CCL20 in rats. Mol. Hum. Reprod. 28 (2), gaac002. 10.1093/molehr/gaac002 35088858

[B191] Maekawa M. Kamimura K. Nagano T. (1996). Peritubular myoid cells in the testis: their structure and function. Arch. Histol. Cytol. 59 (1), 1–13. 10.1679/aohc.59.1 8727359

[B192] Mahyari E. Guo J. Lima A. C. Lewinsohn D. P. Stendahl A. M. Vigh-Conrad K. A. (2021). Comparative single-cell analysis of biopsies clarifies pathogenic mechanisms in Klinefelter syndrome. Am. J. Hum. Genet. 108 (10), 1924–1945. 10.1016/j.ajhg.2021.09.001 34626582 PMC8546046

[B193] Mäkelä J. A. Hobbs R. M. (2019). Molecular regulation of spermatogonial stem cell renewal and differentiation. Reproduction 158 (5), R169–R187. 10.1530/Rep-18-0476 31247585

[B194] Mäkelä J. A. Koskenniemi J. J. Virtanen H. E. Toppari J. (2019). Testis development. Endocr. Rev. 40 (4), 857–905. 10.1210/er.2018-00140 30590466

[B195] Manna P. R. Chandrala S. P. Jo Y. Stocco D. M. (2006). cAMP-independent signaling regulates steroidogenesis in mouse Leydig cells in the absence of StAR phosphorylation. J. Mol. Endocrinol. 37 (1), 81–95. 10.1677/jme.1.02065 16901926

[B196] Mao B. P. Bu T. Mruk D. Li C. Sun F. Cheng C. Y. (2020). Modulating the blood-testis barrier towards increasing drug delivery. Trends Pharmacol. Sci. 41 (10), 690–700. 10.1016/j.tips.2020.07.002 32792159

[B197] Mark M. Teletin M. Vernet N. Ghyselinck N. B. (2015). Role of retinoic acid receptor (RAR) signaling in post-natal male germ cell differentiation. Biochim. Biophys. Acta 1849 (2), 84–93. 10.1016/j.bbagrm.2014.05.019 24875094

[B198] Markouli M. Michala L. (2023). Fertility potential in 5α-reductase type 2 deficient males. J. Pediatr. Urol. 19 (1), 108–114. 10.1016/j.jpurol.2022.09.002 36153242

[B199] Martin L. J. (2016). Cell interactions and genetic regulation that contribute to testicular Leydig cell development and differentiation. Mol. Reprod. Dev. 83 (6), 470–487. 10.1002/mrd.22648 27079813

[B200] Masone M. C. (2023). Embryonal origin of adult testicular macrophages. Nat. Rev. Urol. 20 (5), 262. 10.1038/s41585-023-00770-x 37059869

[B201] Matson C. K. Murphy M. W. Sarver A. L. Griswold M. D. Bardwell V. J. Zarkower D. (2011). DMRT1 prevents female reprogramming in the postnatal mammalian testis. Nature 476 (7358), 101–104. 10.1038/nature10239 21775990 PMC3150961

[B202] Mayerhofer A. (2013). Human testicular peritubular cells: more than meets the eye. Reproduction 145 (5), R107–R116. 10.1530/Rep-12-0497 23431272

[B203] McCabe M. J. Allan C. M. Foo C. F. H. Nicholls P. K. McTavish K. J. Stanton P. G. (2012). Androgen initiates Sertoli cell tight junction formation in the hypogonadal (hpg) mouse. Biol. Reprod. 87 (2), 38. 10.1095/biolreprod.111.094318 22623623

[B204] McCabe M. J. Foo C. F. Dinger M. E. Smooker P. M. Stanton P. G. (2016). Claudin-11 and occludin are major contributors to Sertoli cell tight junction function, *in vitro* . Asian J. Androl. 18 (4), 620–626. 10.4103/1008-682x.163189 26585695 PMC4955190

[B205] McClelland K. S. Bell K. Larney C. Harley V. R. Sinclair A. H. Oshlack A. (2015). Purification and transcriptomic analysis of Mouse Fetal Leydig cells reveals candidate genes for specification of gonadal steroidogenic cells. Biol. Reprod. 92 (6), 145. 10.1095/biolreprod.115.128918 25855264

[B206] McElreavey K. Jorgensen A. Eozenou C. Merel T. Bignon-Topalovic J. Tan D. S. (2020). Pathogenic variants in the DEAH-box RNA helicase DHX37 are a frequent cause of 46,XY gonadal dysgenesis and 46,XY testicular regression syndrome. Genet. Med. 22 (1), 150–159. 10.1038/s41436-019-0606-y 31337883 PMC6944638

[B207] Meachem S. J. Ruwanpura S. M. Ziolkowski J. Ague J. M. Skinner M. K. Loveland K. L. (2005). Developmentally distinct *in vivo* effects of FSH on proliferation and apoptosis during testis maturation. J. Endocrinol. 186 (3), 429–446. 10.1677/joe.1.06121 16135663

[B208] Medici C. Jorgensen N. Juul A. Albrethsen J. Kreiberg M. Lauritsen J. (2024). Insulin-like factor 3, basal and human chorionic gonadotropin-stimulated testosterone as biomarkers to predict the effect of testosterone replacement in testicular cancer survivors with mild Leydig cell insufficiency. Clin. Genitourin. Cancer 22 (1), e106–e112. 10.1016/j.clgc.2023.08.005 37673783

[B209] Meinhardt A. Wang M. Schulz C. Bhushan S. (2018). Microenvironmental signals govern the cellular identity of testicular macrophages. J. Leukoc. Biol. 104 (4), 757–766. 10.1002/Jlb.3mr0318-086rr 30265772

[B210] Meng J. Greenlee A. R. Taub C. J. Braun R. E. (2011). Sertoli cell-specific deletion of the androgen receptor compromises testicular immune privilege in mice. Biol. Reprod. 85 (2), 254–260. 10.1095/biolreprod.110.090621 21543771 PMC3142254

[B211] Meroni S. B. Galardo M. N. Rindone G. Gorga A. Riera M. F. Cigorraga S. B. (2019). Molecular mechanisms and signaling pathways involved in Sertoli cell proliferation. Front. Endocrinol. 10, 224. 10.3389/fendo.2019.00224 31040821 PMC6476933

[B212] Min M. Song T. Sun M. D. Wang T. T. Tan J. Zhang J. D. (2022). Dhh signaling pathway regulates reconstruction of seminiferous tubule-like structure. Reprod. Biol. 22 (4), 100684. 10.1016/j.repbio.2022.100684 35987158

[B213] Mita M. Hall P. F. (1982). Metabolism of round spermatids from rats: lactate as the preferred substrate. Biol. Reprod. 26 (3), 445–455. 10.1095/biolreprod26.3.445 7082719

[B214] Mitra S. Srivastava A. Khandelwal S. (2017). Long term impact of the endocrine disruptor tributyltin on male fertility following a single acute exposure. Environ. Toxicol. 32 (10), 2295–2304. 10.1002/tox.22446 28707438

[B215] Miyabayashi K. Katoh-Fukui Y. Ogawa H. Baba T. Shima Y. Sugiyama N. (2013). Aristaless related homeobox gene, Arx, is implicated in mouse fetal Leydig cell differentiation possibly through expressing in the progenitor cells. Plos One 8 (6), e68050. 10.1371/journal.pone.0068050 23840809 PMC3695952

[B216] Miyamoto Y. Taniguchi H. Hamel F. Silversides D. W. Viger R. S. (2008). A GATA4/WT1 cooperation regulates transcription of genes required for mammalian sex determination and differentiation. Bmc Mol. Biol. 9, 44. 10.1186/1471-2199-9-44 18445271 PMC2387164

[B217] Morianos I. Papadopoulou G. Semitekolou M. Xanthou G. (2019). Activin-A in the regulation of immunity in health and disease. J. Autoimmun. 104, 102314. 10.1016/j.jaut.2019.102314 31416681

[B218] Mossadegh-Keller N. Gentek R. Gimenez G. Bigot S. Mailfert S. Sieweke M. H. (2017). Developmental origin and maintenance of distinct testicular macrophage populations. J. Exp. Med. 214 (10), 2829–2841. 10.1084/jem.20170829 28784628 PMC5626405

[B219] Mruk D. D. Cheng C. Y. (2004). Cell-cell interactions at the ectoplasmic specialization in the testis. Trends Endocrinol. Metab. 15 (9), 439–447. 10.1016/j.tem.2004.09.009 15519891

[B220] Mruk D. D. Cheng C. Y. (2015). The Mammalian blood-testis barrier: its biology and regulation. Endocr. Rev. 36 (5), 564–591. 10.1210/er.2014-1101 26357922 PMC4591527

[B221] Mu Y. Yin T. L. Zhang Y. Yang J. Wu Y. T. (2022). Diet-induced obesity impairs spermatogenesis: the critical role of NLRP3 in Sertoli cells. Inflamm. Regen. 42 (1), 24. 10.1186/s41232-022-00203-z 35915511 PMC9344614

[B222] Murphy C. J. Richburg J. H. (2014). Implications of Sertoli cell induced germ cell apoptosis to testicular pathology. Spermatogenesis 4 (2), e979110. 10.4161/21565562.2014.979110 26413394 PMC4581061

[B223] Nagano R. Tabata S. Nakanishi Y. Ohsako S. Kurohmaru M. Hayashi Y. (2000). Reproliferation and relocation of mouse male germ cells (gonocytes) during prespermatogenesis. Anat. Rec. 258 (2), 210–220. 10.1002/(SICI)1097-0185(20000201)258:2<210::AID-AR10>3.0.CO;2-X 10645968

[B224] Nakagawa A. Shiratsuchi A. Tsuda K. Nakanishi Y. (2005). *In vivo* analysis of phagocytosis of apoptotic cells by testicular Sertoli cells. Mol. Reprod. Dev. 71 (2), 166–177. 10.1002/mrd.20278 15791597

[B225] Nakagawa T. Nabeshima Y. I. Yoshida S. (2007). Functional identification of the actual and potential stem cell compartments in mouse spermatogenesis. Dev. Cell 12 (2), 195–206. 10.1016/j.devcel.2007.01.002 17276338

[B226] Nakamura M. Okinaga S. Arai K. (1984). Metabolism of pachytene primary spermatocytes from rat testes: pyruvate maintenance of adenosine triphosphate level. Biol. Reprod. 30 (5), 1187–1197. 10.1095/biolreprod30.5.1187 6733209

[B227] Naughton C. K. Jain S. Strickland A. M. Gupta A. Milbrandt J. (2006). Glial cell-line derived neurotrophic factor-mediated RET signaling regulates spermatogonial stem cell fate.(vol 74, pg 314, 2006). Biol. Reprod. 75 (4), 660. 10.1095/biolreprod.106.052027 16237148

[B228] Ni F. D. Hao S. L. Yang W. X. (2019). Multiple signaling pathways in Sertoli cells: recent findings in spermatogenesis. Cell Death Dis. 10 (8), 541. 10.1038/s41419-019-1782-z 31316051 PMC6637205

[B229] Ni F. D. Hao S. L. Yang W. X. (2020). Molecular insights into hormone regulation *via* signaling pathways in Sertoli cells: with discussion on infertility and testicular tumor. Gene 753, 144812. 10.1016/j.gene.2020.144812 32470507

[B230] Nicholls P. K. Page D. C. (2021). Germ cell determination and the developmental origin of germ cell tumors. Development 148 (8), dev198150. 10.1242/dev.198150 33913479

[B231] Nie X. Munyoki S. K. Sukhwani M. Schmid N. Missel A. Emery B. R. (2022). Single-cell analysis of human testis aging and correlation with elevated body mass index. Dev. Cell 57 (9), 1160–1176.e1165. 10.1016/j.devcel.2022.04.004 35504286 PMC9090997

[B232] Nistal M. Paniagua R. Regadera J. Santamarìa L. Amat P. (1986). A quantitative morphological study of human Leydig cells from birth to adulthood. Cell Tissue Res. 246 (2), 229–236. 10.1007/bf00215884 3779804

[B233] Niu Q. Cao M. Yuan C. Huang Y. Zhao Z. Zhang B. (2020). Effect of nerve growth factor on the proliferation in newborn bovine testicular Sertoli cells. Reproduction 160 (3), 405–415. 10.1530/rep-19-0601 32567558

[B234] O'Donnell L. (2014). Mechanisms of spermiogenesis and spermiation and how they are disturbed. Spermatogenesis 4 (2), e979623. 10.4161/21565562.2014.979623 26413397 PMC4581055

[B235] O'Donnell L. Nicholls P. K. O'Bryan M. K. McLachlan R. I. Stanton P. G. (2011). Spermiation: the process of sperm release. Spermatogenesis 1 (1), 14–35. 10.4161/spmg.1.1.14525 21866274 PMC3158646

[B236] O'Donnell L. Rebourcet D. Dagley L. F. Sgaier R. Infusini G. O'Shaughnessy P. J. (2021). Sperm proteins and cancer-testis antigens are released by the seminiferous tubules in mice and men. Faseb J. 35 (3), e21397. 10.1096/fj.202002484R 33565176 PMC7898903

[B237] O'Donnell L. Smith L. B. Rebourcet D. (2022a). Sertoli cells as key drivers of testis function. Seminars Cell and Dev. Biol. 121, 2–9. 10.1016/j.semcdb.2021.06.016 34229950

[B238] O'Donnell L. Whiley P. A. F. Loveland K. L. (2022b). Activin A and sertoli cells: key to fetal testis steroidogenesis. Front. Endocrinol. (Lausanne) 13, 898876. 10.3389/fendo.2022.898876 35685219 PMC9171382

[B239] O'Shaughnessy P. J. Johnston H. Willerton L. Baker P. J. (2002). Failure of normal adult Leydig cell development in androgen-receptor-deficient mice. J. Cell Sci. 115 (17), 3491–3496. 10.1242/jcs.115.17.3491 12154079

[B240] Oatley J. M. Brinster R. L. (2012). The germline stem cell niche unit in Mammalian testes. Physiol. Rev. 92 (2), 577–595. 10.1152/physrev.00025.2011 22535892 PMC3970841

[B241] Oatley M. J. Racicot K. E. Oatley J. M. (2011). Sertoli cells dictate spermatogonial stem cell niches in the mouse testis. Biol. Reprod. 84 (4), 639–645. 10.1095/biolreprod.110.087320 21084712 PMC3062034

[B242] Oduwole O. O. Vydra N. Wood N. E. M. Samanta L. Owen L. Keevil B. (2014). Overlapping dose responses of spermatogenic and extragonadal testosterone actions jeopardize the principle of hormonal male contraception. Faseb J. 28 (6), 2566–2576. 10.1096/fj.13-249219 24599970 PMC4376501

[B243] Okashita N. Tachibana M. (2021). Transcriptional regulation of the Y-Linked Mammalian testis-determining gene SRY. Sex. Dev. 15 (5-6), 351–359. 10.1159/000519217 34583357

[B244] Oliveira P. F. Alves M. G. Rato L. Laurentino S. Silva J. Sá R. (2012). Effect of insulin deprivation on metabolism and metabolism-associated gene transcript levels of *in vitro* cultured human Sertoli cells. Biochim. Biophys. Acta-General Subj. 1820 (2), 84–89. 10.1016/j.bbagen.2011.11.006 22146232

[B245] Oonk R. B. Grootegoed J. A. van der Molen H. J. (1985). Comparison of the effects of insulin and follitropin on glucose metabolism by Sertoli cells from immature rats. Mol. Cell Endocrinol. 42 (1), 39–48. 10.1016/0303-7207(85)90005-x 3928417

[B246] Otake S. Park M. K. (2016). Expressional changes of AMH signaling system in the quail testis induced by photoperiod. Reproduction 152 (5), 575–589. 10.1530/Rep-16-0175 27581082

[B247] Palmer S. J. Burgoyne P. S. (1991). *In situ* analysis of fetal, prepuberal and adult XX----XY chimaeric mouse testes: sertoli cells are predominantly, but not exclusively, XY. Development 112 (1), 265–268. 10.1242/dev.112.1.265 1769333

[B248] Paniagua R. Nistal M. Amat P. Rodriguez M. C. Martin A. (1987). Seminiferous tubule involution in elderly men. Biol. Reprod. 36 (4), 939–947. 10.1095/biolreprod36.4.939 3593859

[B249] Parekh P. A. Garcia T. X. Hofmann M. C. (2019). Regulation of GDNF expression in Sertoli cells. Reproduction 157 (3), R95–R107. 10.1530/REP-18-0239 30620720 PMC6602878

[B250] Park S. Y. Kim I. S. (2019). Stabilin receptors: role as phosphatidylserine receptors. Biomolecules 9 (8), 387. 10.3390/biom9080387 31434355 PMC6723754

[B251] Park H. J. Lee W. Y. Do J. T. Park C. Song H. (2021). Evaluation of testicular toxicity upon fetal exposure to bisphenol A using an organ culture method. Chemosphere 270, 129445. 10.1016/j.chemosphere.2020.129445 33421752

[B252] Patrikidou A. Cazzaniga W. Berney D. Boormans J. de Angst I. Di Nardo D. (2023). European Association of Urology Guidelines on testicular cancer: 2023 update. Eur. Urol. 84 (3), 289–301. 10.1016/j.eururo.2023.04.010 37183161

[B253] Peirouvi T. Aliaghaei A. Eslami Farsani B. Ziaeipour S. Ebrahimi V. Forozesh M. (2021). COVID-19 disrupts the blood-testis barrier through the induction of inflammatory cytokines and disruption of junctional proteins. Inflamm. Res. 70 (10-12), 1165–1175. 10.1007/s00011-021-01497-4 34436630 PMC8387554

[B254] Pérez C. V. Theas M. S. Jacobo P. V. Jarazo-Dietrich S. Guazzone V. A. Lustig L. (2013). Dual role of immune cells in the testis: protective or pathogenic for germ cells? Spermatogenesis 3 (1), e23870. 10.4161/spmg.23870 23687616 PMC3644047

[B255] Pierucci-Alves F. Clark A. M. Russell L. D. (2001). A developmental study of the Desert hedgehog-null mouse testis. Biol. Reprod. 65 (5), 1392–1402. 10.1095/biolreprod65.5.1392 11673255

[B256] Pointis G. Gilleron J. Carette D. Segretain D. (2010). Physiological and physiopathological aspects of connexins and communicating gap junctions in spermatogenesis. Philos. Trans. R. Soc. B-Biological Sci. 365 (1546), 1607–1620. 10.1098/rstb.2009.0114 20403873 PMC2871914

[B257] Potter S. J. DeFalco T. (2017). Role of the testis interstitial compartment in spermatogonial stem cell function. Reproduction 153 (4), R151–R162. 10.1530/REP-16-0588 28115580 PMC5326597

[B258] Poulsen K. H. Nielsen J. E. Frederiksen H. Melau C. Hare K. J. Thuesen L. L. (2019). Dysregulation of FGFR signalling by a selective inhibitor reduces germ cell survival in human fetal gonads of both sexes and alters the somatic niche in fetal testes. Hum. Reprod. 34 (11), 2228–2243. 10.1093/humrep/dez191 31734698 PMC6994936

[B259] Prince F. P. (1990). Ultrastructural evidence of mature Leydig cells and Leydig cell regression in the neonatal human testis. Anat. Rec. 228 (4), 405–417. 10.1002/ar.1092280406 2178325

[B260] Prince F. P. (1992). Ultrastructural evidence of indirect and direct autonomic innervation of human Leydig-Cells - comparison of neonatal, childhood and pubertal ages. Cell Tissue Res. 269 (3), 383–390. 10.1007/Bf00353893 1423506

[B261] Prince F. P. (2001). The triphasic nature of Leydig cell development in humans, and comments on nomenclature. J. Endocrinol. 168 (2), 213–216. 10.1677/joe.0.1680213 11182757

[B262] Qi S. Fu W. Wang C. Liu C. Quan C. Kourouma A. (2014). BPA-induced apoptosis of rat Sertoli cells through Fas/FasL and JNKs/p38 MAPK pathways. Reprod. Toxicol. 50, 108–116. 10.1016/j.reprotox.2014.10.013 25461909

[B263] Qian Y. Liu S. J. Guan Y. T. Pan H. J. Guan X. Qiu Z. W. (2013). Lgr4-mediated Wnt/β-catenin signaling in peritubular myoid cells is essential for spermatogenesis. Development 140 (8), 1751–1761. 10.1242/dev.093641 23533175

[B264] Qian X. J. Mruk D. D. Cheng Y. H. Tang E. I. Han D. S. Lee W. M. (2014). Actin binding proteins, spermatid transport and spermiation. Seminars Cell and Dev. Biol. 30, 75–85. 10.1016/j.semcdb.2014.04.018 24735648 PMC4063300

[B265] Qian G. Q. Wang X. C. Zhang X. Shen B. Liu Q. (2023). Pyruvate kinase M in germ cells is essential for sperm motility and male fertility but not spermatogenesis. Asian J. Androl. 26 (2), 212–219. 10.4103/aja202350 37902871 PMC10919421

[B266] Rajachandran S. Zhang X. Cao Q. Caldeira-Brant A. L. Zhang X. Song Y. (2023). Dissecting the spermatogonial stem cell niche using spatial transcriptomics. Cell Rep. 42 (7), 112737. 10.1016/j.celrep.2023.112737 37393620 PMC10530051

[B267] Raverdeau M. Gely-Pernot A. Féret B. Dennefeld C. Benoit G. Davidson I. (2012). Retinoic acid induces Sertoli cell paracrine signals for spermatogonia differentiation but cell autonomously drives spermatocyte meiosis. Proc. Natl. Acad. Sci. U. S. A. 109 (41), 16582–16587. 10.1073/pnas.1214936109 23012458 PMC3478620

[B268] Rebourcet D. O'Shaughnessy P. J. Monteiro A. Milne L. Cruickshanks L. Jeffrey N. (2014a). Sertoli cells maintain Leydig cell number and peritubular myoid cell activity in the adult mouse testis. Plos One 9 (8), e105687. 10.1371/journal.pone.0105687 25144714 PMC4140823

[B269] Rebourcet D. O'Shaughnessy P. J. Pitetti J. L. Monteiro A. O'Hara L. Milne L. (2014b). Sertoli cells control peritubular myoid cell fate and support adult Leydig cell development in the prepubertal testis. Development 141 (10), 2139–2149. 10.1242/dev.107029 24803659 PMC4011090

[B270] Rebourcet D. Darbey A. Monteiro A. Soffientini U. Tsai Y. T. Handel I. (2017). Sertoli cell number defines and predicts germ and Leydig cell population sizes in the adult mouse testis. Endocrinology 158 (9), 2955–2969. 10.1210/en.2017-00196 28911170 PMC5659676

[B271] Regueira M. Gorga A. Rindone G. M. Pellizzari E. H. Cigorraga S. B. Galardo M. N. (2018). Apoptotic germ cells regulate Sertoli cell lipid storage and fatty acid oxidation. Reproduction 156 (6), 515–525. 10.1530/rep-18-0181 30328346

[B272] Ren F. Fang Q. Xi H. M. Feng T. Y. Wang L. Q. Hu J. H. (2020). Platelet-derived growth factor-BB and epidermal growth factor promote dairy goat spermatogonial stem cells proliferation *via* Ras/ERK1/2 signaling pathway. Theriogenology 155, 205–212. 10.1016/j.theriogenology.2020.06.012 32721699

[B273] Ren M. D. Xu Y. Phoon C. K. L. Erdjument-Bromage H. Neubert T. A. Rajan S. (2022). Condensed mitochondria assemble into the acrosomal matrix during spermiogenesis. Front. Cell Dev. Biol. 10, 867175. 10.3389/fcell.2022.867175 35531097 PMC9068883

[B274] Richardson B. E. Lehmann R. (2010). Mechanisms guiding primordial germ cell migration: strategies from different organisms. Nat. Rev. Mol. Cell Biol. 11 (1), 37–49. 10.1038/nrm2815 20027186 PMC4521894

[B275] Richardson L. L. Kleinman H. K. Dym M. (1995). Basement-Membrane gene-expression by Sertoli and peritubular myoid cells *in-vitro* in the rat. Biol. Reprod. 52 (2), 320–330. 10.1095/biolreprod52.2.320 7711202

[B276] Robinson M. Haegert A. Li Y. Y. Morova T. Zhang A. Y. Y. Witherspoon L. (2023). Differentiation of peritubular myoid-like cells from human induced pluripotent stem cells. Adv. Biol. 7 (7), e2200322. 10.1002/adbi.202200322 36895072

[B277] Rodriguez K. F. Brown P. R. Amato C. M. Nicol B. Liu C. F. Xu X. (2022). Somatic cell fate maintenance in mouse fetal testes *via* autocrine/paracrine action of AMH and activin B. Nat. Commun. 13 (1), 4130. 10.1038/s41467-022-31486-y 35840551 PMC9287316

[B278] Rossi F. Guerrini L. Pasimeni G. Markouizou A. Fabbrini A. Santiemma V. (2000). Testicular peritubular myoid cells are a target for adrenomedullin. Arch. Androl. 44 (2), 103–107. 10.1080/014850100262263 10746866

[B279] Rossi G. Dufrusine B. Lizzi A. R. Luzi C. Piccoli A. Fezza F. (2020). Bisphenol A deranges the endocannabinoid System of primary sertoli cells with an impact on inhibin B production. Int. J. Mol. Sci. 21 (23), 8986. 10.3390/ijms21238986 33256105 PMC7730056

[B280] Rotgers E. Jorgensen A. Yao H. H. (2018). At the crossroads of fate-somatic cell lineage specification in the fetal gonad. Endocr. Rev. 39 (5), 739–759. 10.1210/er.2018-00010 29771299 PMC6173476

[B281] Roth D. M. Bayona F. Baddam P. Graf D. (2021). Craniofacial development: neural crest in molecular embryology. Head. Neck Pathol. 15 (1), 1–15. 10.1007/s12105-021-01301-z 33723764 PMC8010074

[B282] Ruwanpura S. M. McLachlan R. I. Meachem S. J. (2010). Hormonal regulation of male germ cell development. J. Endocrinol. 205 (2), 117–131. 10.1677/Joe-10-0025 20144980

[B283] Ryu B. Y. Orwig K. E. Oatley J. M. Avarbock M. R. Brinster R. L. (2006). Effects of aging and niche microenvironment on spermatogonial stem cell self-renewal. Stem Cells 24 (6), 1505–1511. 10.1634/stemcells.2005-0580 16456131 PMC5501308

[B284] Saez J. M. Avallet O. Naville D. Perrard-Sapori M. H. Chatelain P. G. (1989). Sertoli-Leydig cell communications. Ann. N. Y. Acad. Sci. 564, 210–231. 10.1111/j.1749-6632.1989.tb25899.x 2505656

[B285] Saitou M. Miyauchi H. (2016). Gametogenesis from pluripotent stem cells. Cell Stem Cell 18 (6), 721–735. 10.1016/j.stem.2016.05.001 27257761

[B286] Saitou M. Yamaji M. (2010). Germ cell specification in mice: signaling, transcription regulation, and epigenetic consequences. Reproduction 139 (6), 931–942. 10.1530/Rep-10-0043 20371640

[B287] Saitou M. Yamaji M. (2012). Primordial germ cells in mice. Cold Spring Harb. Perspect. Biol. 4 (11), a008375. 10.1101/cshperspect.a008375 23125014 PMC3536339

[B288] Sandell L. L. Lynn M. L. Inman K. E. McDowell W. Trainor P. A. (2012). RDH10 oxidation of vitamin A is a critical control step in synthesis of retinoic acid during Mouse embryogenesis. Plos One 7 (2), e30698. 10.1371/journal.pone.0030698 22319578 PMC3271098

[B289] Santiago J. Silva J. V. Alves M. G. Oliveira P. F. Fardilha M. (2019). Testicular aging: an overview of ultrastructural, cellular, and molecular alterations. J. Gerontol. A Biol. Sci. Med. Sci. 74 (6), 860–871. 10.1093/gerona/gly082 29688289

[B290] Saracino R. Capponi C. Di Persio S. Boitani C. Masciarelli S. Fazi F. (2020). Regulation of Gdnf expression by retinoic acid in Sertoli cells. Mol. Reprod. Dev. 87 (4), 419–429. 10.1002/mrd.23323 32020743

[B291] Sarkar O. Mathur P. P. Cheng C. Y. Mruk D. D. (2008). Interleukin 1 alpha (IL1A) is a novel regulator of the blood-testis barrier in the rat. Biol. Reprod. 78 (3), 445–454. 10.1095/biolreprod.107.064501 18057314 PMC2804918

[B292] Sasaki K. Nakamura T. Okamoto K. Yabuta Y. Iwatani C. Tsuchiya H. (2016). The germ cell fate of cynomolgus monkeys is specified in the nascent amnion. Dev. Cell 39 (2), 169–185. 10.1016/j.devcel.2016.09.007 27720607

[B293] Sawaied A. Levy B. E. Arazi E. Lunenfeld E. Shi Q. Huleihel M. (2025). Follicle-Stimulating hormone and testosterone play a role in the regulation of Sertoli cell functions following germ cell depletion *in vitro* . Int. J. Mol. Sci. 26 (6), 2702. 10.3390/ijms26062702 40141344 PMC11942298

[B294] Schmahl J. Eicher E. M. Washburn L. L. Capel B. (2000). Sry induces cell proliferation in the mouse gonad. Development 127 (1), 65–73. 10.1242/dev.127.1.65 10654601

[B295] Scobey M. Bertera S. Somers J. Watkins S. Zeleznik A. Walker W. (2001). Delivery of a cyclic adenosine 3',5'-monophosphate response element-binding protein (creb) mutant to seminiferous tubules results in impaired spermatogenesis. Endocrinology 142 (2), 948–954. 10.1210/endo.142.2.7948 11159868

[B296] Seki Y. Yamaji M. Yabuta Y. Sano M. Shigeta M. Matsui Y. (2007). Cellular dynamics associated with the genome-wide epigenetic reprogramming in migrating primordial germ cells in mice. Development 134 (14), 2627–2638. 10.1242/dev.005611 17567665

[B297] Sekido R. Lovell-Badge R. (2008). Sex determination involves synergistic action of SRY and SF1 on a specific Sox9 enhancer. Nature 453 (7197), 930–934. 10.1038/nature06944 18454134

[B298] Sekido R. Lovell-Badge R. (2013). Genetic control of Testis development. Sex. Dev. 7 (1-3), 21–32. 10.1159/000342221 22964823

[B299] Sharpe R. M. McKinnell C. Kivlin C. Fisher J. S. (2003). Proliferation and functional maturation of Sertoli cells, and their relevance to disorders of testis function in adulthood. Reproduction 125 (6), 769–784. 10.1530/rep.0.1250769 12773099

[B300] Shima Y. (2019). Development of fetal and adult Leydig cells. Reprod. Med. Biol. 18 (4), 323–330. 10.1002/rmb2.12287 31607792 PMC6780029

[B301] Shima Y. Miyabayashi K. Haraguchi S. Arakawa T. Otake H. Baba T. (2013). Contribution of Leydig and Sertoli cells to testosterone production in Mouse fetal testes. Mol. Endocrinol. 27 (1), 63–73. 10.1210/me.2012-1256 23125070 PMC5416943

[B302] Shima Y. Matsuzaki S. Miyabayashi K. Otake H. Baba T. Kato S. (2015). Fetal Leydig cells persist as an androgen-independent subpopulation in the postnatal testis. Mol. Endocrinol. 29 (11), 1581–1593. 10.1210/me.2015-1200 26402718 PMC5414671

[B303] Shima Y. Miyabayashi K. Sato T. Suyama M. Ohkawa Y. Doi M. (2018). Fetal Leydig cells dedifferentiate and serve as adult Leydig stem cells. Development 145 (23), dev169136. 10.1242/dev.169136 30518625

[B304] Skakkebæk N. E. Lindahl-Jacobsen R. Levine H. Andersson A. M. Jorgensen N. Main K. M. (2022). Environmental factors in declining human fertility. Nat. Rev. Endocrinol. 18 (3), 139–157. 10.1038/s41574-021-00598-8 34912078

[B305] Skinner M. K. Griswold M. D. (1983). Sertoli cells synthesize and secrete a ceruloplasmin-like protein. Biol. Reprod. 28 (5), 1225–1229. 10.1095/biolreprod28.5.1225 6871315

[B306] Smith L. B. Walker W. H. (2014). The regulation of spermatogenesis by androgens. Seminars Cell and Dev. Biol. 30, 2–13. 10.1016/j.semcdb.2014.02.012 24598768 PMC4043871

[B307] Stanton P. G. Sluka P. Foo C. F. H. Stephens A. N. Smith A. I. McLachlan R. I. (2012). Proteomic changes in rat spermatogenesis in response to *in vivo* androgen manipulation; impact on meiotic cells. Plos One 7 (7), e41718. 10.1371/journal.pone.0041718 22860010 PMC3408499

[B308] Stukenborg J. B. Mitchell R. T. Soder O. (2021). Endocrine disruptors and the male reproductive system. Best. Pract. Res. Clin. Endocrinol. Metab. 35 (5), 101567. 10.1016/j.beem.2021.101567 34426080

[B309] Su J. Yang Y. Wang D. Su H. Zhao F. Zhang C. (2025). A dynamic transcriptional cell atlas of testes development after birth in Hu sheep. BMC Biol. 23 (1), 78. 10.1186/s12915-025-02186-y 40075363 PMC11905504

[B310] Sumaiya K. Langford D. Natarajaseenivasan K. Shanmughapriya S. (2022). Macrophage migration inhibitory factor (MIF): a multifaceted cytokine regulated by genetic and physiological strategies. Pharmacol. Ther. 233, 108024. 10.1016/j.pharmthera.2021.108024 34673115

[B311] Svingen T. Koopman P. (2013). Building the mammalian testis: origins, differentiation, and assembly of the component cell populations. Genes and Dev. 27 (22), 2409–2426. 10.1101/gad.228080.113 24240231 PMC3841730

[B312] Tan K. Song H. W. Wilkinson M. F. (2020). Single-cell RNAseq analysis of testicular germ and somatic cell development during the perinatal period. Development 147 (3), dev183251. 10.1242/dev.183251 31964773 PMC7033731

[B313] Tanphaichitr N. Kongmanas K. Faull K. F. Whitelegge J. Compostella F. Goto-Inoue N. (2018). Properties, metabolism and roles of sulfogalactosylglycerolipid in male reproduction. Prog. Lipid Res. 72, 18–41. 10.1016/j.plipres.2018.08.002 30149090 PMC6239905

[B314] Tao H. P. Lu T. F. Li S. Jia G. X. Zhang X. N. Yang Q. E. (2023). Pancreatic lipase-related protein 2 is selectively expressed by peritubular myoid cells in the murine testis and sustains long-term spermatogenesis. Cell Mol. Life Sci. 80 (8), 217. 10.1007/s00018-023-04872-y 37468762 PMC11072130

[B315] Teerds K. J. Huhtaniemi I. T. (2015). Morphological and functional maturation of Leydig cells: from rodent models to primates. Hum. Reprod. Update 21 (3), 310–328. 10.1093/humupd/dmv008 25724971

[B316] Teletin M. Vernet N. Yu J. S. Klopfenstein M. Jones J. W. Féret B. (2019). Two functionally redundant sources of retinoic acid secure spermatogonia differentiation in the seminiferous epithelium. Development 146 (1), dev170225. 10.1242/dev.170225 30487180 PMC6340151

[B317] Tevosian S. G. Albrecht K. H. Crispino J. D. Fujiwara Y. Eicher E. M. Orkin S. H. (2002). Gonadal differentiation, sex determination and normal Sry expression in mice require direct interaction between transcription partners GATA4 and FOG2. Development 129 (19), 4627–4634. 10.1242/dev.129.19.4627 12223418

[B318] Theodosiou M. Laudet V. Schubert M. (2010). From carrot to clinic: an overview of the retinoic acid signaling pathway. Cell. Mol. Life Sci. 67 (9), 1423–1445. 10.1007/s00018-010-0268-z 20140749 PMC11115864

[B319] Tian R. Yao C. Yang C. Zhu Z. Li C. Zhi E. (2019). Fibroblast growth factor-5 promotes spermatogonial stem cell proliferation *via* ERK and AKT activation. Stem Cell Res. Ther. 10 (1), 40. 10.1186/s13287-019-1139-7 30670081 PMC6343348

[B320] Tong M. H. Yang Q. E. Davis J. C. Griswold M. D. (2013). Retinol dehydrogenase 10 is indispensible for spermatogenesis in juvenile males. Proc. Natl. Acad. Sci. U. S. A. 110 (2), 543–548. 10.1073/pnas.1214883110 23267101 PMC3545805

[B321] Travison T. G. Araujo A. B. Hall S. A. McKinlay J. B. (2009). Temporal trends in testosterone levels and treatment in older men. Curr. Opin. Endocrinol. Diabetes Obes. 16 (3), 211–217. 10.1097/MED.0b013e32832b6348 19396984

[B322] Tsikolia N. Merkwitz C. Sass K. Sakurai M. Spanel-Borowski K. Ricken A. M. (2009). Characterization of bovine fetal Leydig cells by KIT expression. Histochem. Cell Biol. 132 (6), 623–632. 10.1007/s00418-009-0640-y 19768462

[B323] Tsuji-Hosokawa A. Kashimada K. Kato T. Ogawa Y. Nomura R. Takasawa K. (2018). Peptidyl arginine deiminase 2 (Padi2) is expressed in Sertoli cells in a specific manner and regulated by SOX9 during testicular development. Sci. Rep. 8, 13263. 10.1038/s41598-018-31376-8 30185873 PMC6125343

[B324] Tung K. S. Harakal J. Qiao H. Rival C. Li J. C. Paul A. G. (2017). Egress of sperm autoantigen from seminiferous tubules maintains systemic tolerance. J. Clin. Invest 127 (3), 1046–1060. 10.1172/jci89927 28218625 PMC5330742

[B325] Tysoe O. (2024). Sertoli cell lysosomal dysfunction drives age-related testicular degeneration. Nat. Rev. Endocrinol. 20 (7), 386. 10.1038/s41574-024-01001-y 38778093

[B326] Uchida A. Sakib S. Labit E. Abbasi S. Scott R. W. Underhill T. M. (2020). Development and function of smooth muscle cells is modulated by *Hic1* in mouse testis. Development 147 (13), dev185884. 10.1242/dev.185884 32554530 PMC7375483

[B327] Val P. Lefrançois-Martinez A. M. Veyssière G. Martinez A. (2003). SF-1 a key player in the development and differentiation of steroidogenic tissues. Nucl. Recept 1 (1), 8. 10.1186/1478-1336-1-8 14594453 PMC240021

[B328] van Pelt A. M. de Rooij D. G. (1990). Synchronization of the seminiferous epithelium after vitamin A replacement in vitamin A-deficient mice. Biol. Reprod. 43 (3), 363–367. 10.1095/biolreprod43.3.363 2271719

[B329] Venditti M. Ben Rhouma M. Romano M. Z. Messaoudi I. Reiter R. J. Minucci S. (2021). Evidence of melatonin ameliorative effects on the blood-testis barrier and sperm quality alterations induced by cadmium in the rat testis. Ecotoxicol. Environ. Saf. 226, 112878. 10.1016/j.ecoenv.2021.112878 34634736

[B330] Vernet N. Dennefeld C. Guillou F. Chambon P. Ghyselinck N. B. Mark M. (2006). Prepubertal testis development relies on retinoic acid but not rexinoid receptors in Sertoli cells. Embo J. 25 (24), 5816–5825. 10.1038/sj.emboj.7601447 17124491 PMC1698894

[B331] Vernet N. Dennefeld C. Klopfenstein M. Ruiz A. Bok D. Ghyselinck N. B. (2008). Retinoid X receptor beta (RXRB) expression in Sertoli cells controls cholesterol homeostasis and spermiation. Reproduction 136 (5), 619–626. 10.1530/Rep-08-0235 18713813

[B332] Wakayama T. Nakata H. Kumchantuek T. Gewaily M. S. Iseki S. (2015). Identification of 5-bromo-2'-deoxyuridine-labeled cells during mouse spermatogenesis by heat-induced antigen retrieval in lectin staining and immunohistochemistry. J. Histochem Cytochem 63 (3), 190–205. 10.1369/0022155414564870 25479790 PMC4340735

[B333] Walker W. H. (2010). Non-classical actions of testosterone and spermatogenesis. Philos. Trans. R. Soc. B-Biological Sci. 365 (1546), 1557–1569. 10.1098/rstb.2009.0258 20403869 PMC2871922

[B334] Wan H. T. Mruk D. D. Wong C. K. C. Cheng C. Y. (2013). The apical ES-BTB-BM functional axis is an emerging target for toxicant-induced infertility. Trends Mol. Med. 19 (7), 396–405. 10.1016/j.molmed.2013.03.006 23643465 PMC3699959

[B335] Wang X. N. Li Z. S. Ren Y. Jiang T. Wang Y. Q. Chen M. (2013). The Wilms tumor gene, Wt1, is critical for mouse spermatogenesis *via* regulation of sertoli cell polarity and is associated with non-obstructive azoospermia in humans. PLoS Genet. 9 (8), e1003645. 10.1371/journal.pgen.1003645 23935527 PMC3731222

[B336] Wang Y. Q. Batool A. Chen S. R. Liu Y. X. (2018a). GATA4 is a negative regulator of contractility in mouse testicular peritubular myoid cells. Reproduction 156 (4), 343–351–351. 10.1530/Rep-18-0148 30306767

[B337] Wang Y. Q. Chen S. R. Liu Y. X. (2018b). Selective deletion of WLS in peritubular myoid cells does not affect spermatogenesis or fertility in mice. Mol. Reprod. Dev. 85 (7), 559–561. 10.1002/mrd.22988 29693774

[B338] Wang M. Yang Y. L. Cansever D. Wang Y. M. Kantores C. Messiaen S. (2021). Two populations of self-maintaining monocyte-independent macrophages exist in adult epididymis and testis. Proc. Natl. Acad. Sci. U. S. A. 118 (1), e2013686117. 10.1073/pnas.2013686117 33372158 PMC7817195

[B339] Wang J. M. Li Z. F. Yang W. X. Tan F. Q. (2022). Follicle-stimulating hormone signaling in Sertoli cells: a licence to the early stages of spermatogenesis. Reprod. Biol. Endocrinol. 20 (1), 97. 10.1186/s12958-022-00971-w 35780146 PMC9250200

[B340] Wang X. Liu X. Qu M. Li H. (2023a). Sertoli cell-only syndrome: advances, challenges, and perspectives in genetics and mechanisms. Cell Mol. Life Sci. 80 (3), 67. 10.1007/s00018-023-04723-w 36814036 PMC11072804

[B341] Wang X. Pei J. Xiong L. Guo S. Cao M. Kang Y. (2023b). Single-Cell RNA sequencing reveals Atlas of yak testis cells. Int. J. Mol. Sci. 24 (9), 7982. 10.3390/ijms24097982 37175687 PMC10178277

[B342] Wang X. Wang Y. Wang Y. Guo Y. Zong R. Hu S. (2024). Single-cell transcriptomic and cross-species comparison analyses reveal distinct molecular changes of porcine testes during puberty. Commun. Biol. 7 (1), 1478. 10.1038/s42003-024-07163-9 39521938 PMC11550399

[B343] Wanjari U. R. Gopalakrishnan A. V. (2024). Blood-testis barrier: a review on regulators in maintaining cell junction integrity between Sertoli cells. Cell Tissue Res. 396 (2), 157–175. 10.1007/s00441-024-03894-7 38564020

[B344] Warr N. Carre G. A. Siggers P. Faleato J. V. Brixey R. Pope M. (2012). Gadd45γ and Map3k4 interactions regulate mouse testis determination *via* p38 MAPK-mediated control of Sry expression. Dev. Cell 23 (5), 1020–1031. 10.1016/j.devcel.2012.09.016 23102580 PMC3526779

[B345] Washburn R. L. Dufour J. M. (2023). Complementing testicular immune regulation: the relationship between Sertoli cells, complement, and the immune response. Int. J. Mol. Sci. 24 (4), 3371. 10.3390/ijms24043371 36834786 PMC9965741

[B346] Wen Q. Zheng Q. S. Li X. X. Hu Z. Y. Gao F. Cheng C. Y. (2014). Wt1 dictates the fate of fetal and adult Leydig cells during development in the mouse testis. Am. J. Physiology-Endocrinology Metab. 307 (12), E1131–E1143. 10.1152/ajpendo.00425.2014 25336526 PMC6189632

[B347] Wen Q. Wang Y. Tang J. Cheng C. Y. Liu Y. X. (2016). Sertoli cell Wt1 regulates peritubular myoid cell and fetal Leydig cell differentiation during fetal Testis development. PLoS One 11 (12), e0167920. 10.1371/journal.pone.0167920 28036337 PMC5201236

[B348] Western P. S. Miles D. C. van den Bergen J. A. Burton M. Sinclair A. H. (2008). Dynamic regulation of mitotic arrest in fetal male germ cells. Stem Cells 26 (2), 339–347. 10.1634/stemcells.2007-0622 18024419

[B349] Wijayarathna R. de Kretser D. M. (2016). Activins in reproductive biology and beyond. Hum. Reprod. Update 22 (3), 342–357. 10.1093/humupd/dmv058 26884470

[B350] Wilhelm D. Englert C. (2002). The Wilms tumor suppressor WT1 regulates early gonad development by activation of Sf1. Genes Dev. 16 (14), 1839–1851. 10.1101/gad.220102 12130543 PMC186395

[B351] Willems A. De Gendt K. Allemeersch J. Smith L. B. Welsh M. Swinnen J. V. (2010). Early effects of Sertoli cell-selective androgen receptor ablation on testicular gene expression. Int. J. Androl. 33 (3), 507–517. 10.1111/j.1365-2605.2009.00964.x 19392831

[B352] Winge S. B. Soraggi S. Schierup M. H. Rajpert-De Meyts E. Almstrup K. (2020). Integration and reanalysis of transcriptomics and methylomics data derived from blood and testis tissue of men with 47,XXY Klinefelter syndrome indicates the primary involvement of Sertoli cells in the testicular pathogenesis. Am. J. Med. Genet. C Semin. Med. Genet. 184 (2), 239–255. 10.1002/ajmg.c.31793 32449318

[B353] Wong C. H. Mruk D. D. Lui W. Y. Cheng C. Y. (2004). Regulation of blood-testis barrier dynamics: an *in vivo* study. J. Cell Sci. 117 (5), 783–798. 10.1242/jcs.00900 14734653

[B354] Wu Q. Y. Shui Y. C. Xia X. Y. Huang Y. F. (2015). FSH and FSHR gene polymorphisms and male infertility: an update. Zhonghua Nan Ke Xue 21 (11), 1031–1034. 10.13263/j.cnki.nja.2015.11.015 26738333

[B355] Xia W. L. Wong E. W. P. Mruk D. D. Cheng C. Y. (2009). TGF-β3 and TNFalpha perturb blood-testis barrier (BTB) dynamics by accelerating the clathrin-mediated endocytosis of integral membrane proteins: a new concept of BTB regulation during spermatogenesis. Dev. Biol. 327 (1), 48–61. 10.1016/j.ydbio.2008.11.028 19103189 PMC2758298

[B356] Xiao Z. Liang J. Huang R. Chen D. Mei J. Deng J. (2024). Inhibition of miR-143-3p restores blood-testis barrier function and ameliorates sertoli cell senescence. Cells 13 (4), 313. 10.3390/cells13040313 38391926 PMC10887369

[B357] Xiong Z. Wang C. Wang Z. Dai H. Song Q. Zou Z. (2018). Raptor directs Sertoli cell cytoskeletal organization and polarity in the mouse testis. Biol. Reprod. 99 (6), 1289–1302. 10.1093/biolre/ioy144 29961810

[B358] Xu Q. Chen H. (2025). Applications of spatial transcriptomics in studying spermatogenesis. Andrology 13, 1181–1189. 10.1111/andr.70043 40202007 PMC12183010

[B359] Xu C. Mohsin A. Luo Y. Xie L. Peng Y. Wang Q. (2020). Inducing non-genetically modified induced embryonic sertoli cells derived from embryonic stem cells with recombinant protein factors. Front. Cell Dev. Biol. 8, 533543. 10.3389/fcell.2020.533543 33585437 PMC7875124

[B360] Yamashita Y. M. (2020). When the family treasure is a doormat. Dev. Cell 52 (1), 3–4. 10.1016/j.devcel.2019.12.013 31951554

[B361] Yan Z. Wang P. Yang Q. Gun S. (2024). Single-Cell RNA sequencing reveals an Atlas of Hezuo pig Testis cells. Int. J. Mol. Sci. 25 (18), 9786. 10.3390/ijms25189786 39337274 PMC11431743

[B362] Yang C. Yao C. Tian R. Zhu Z. Zhao L. Li P. (2019). miR-202-3p regulates sertoli cell proliferation, synthesis function, and apoptosis by targeting LRP6 and Cyclin D1 of Wnt/β-Catenin signaling. Mol. Ther. Nucleic Acids 14, 1–19. 10.1016/j.omtn.2018.10.012 30513418 PMC6280020

[B363] Yang F. Whelan E. C. Guan X. B. Deng B. Q. Wang S. Sun J. C. (2021). FGF9 promotes mouse spermatogonial stem cell proliferation mediated by p38 MAPK signalling. Cell Prolif. 54 (1), e12933. 10.1111/cpr.12933 33107118 PMC7791179

[B364] Yang Y. Yao M. Zeng J. Zheng D. Li Q. Ni Y. (2022). FYN regulates cell adhesion at the blood-testis barrier and the apical ectoplasmic specialization *via* its effect on Arp3 in the mouse testis. Front. Immunol. 13, 915274. 10.3389/fimmu.2022.915274 36016954 PMC9396411

[B365] Yao H. H. C. Whoriskey W. Capel B. (2002). Desert Hedgehog/Patched 1 signaling specifies fetal Leydig cell fate in testis organogenesis. Genes and Dev. 16 (11), 1433–1440. 10.1101/gad.981202 12050120 PMC186321

[B366] Yefimova M. G. Sow A. Fontaine I. Guilleminot V. Martinat N. Crepieux P. (2008). Dimeric transferrin inhibits phagocytosis of residual bodies by testicular rat Sertoli cells. Biol. Reprod. 78 (4), 697–704. 10.1095/biolreprod.107.063107 18094362

[B367] Yefimova M. G. Messaddeq N. Meunier A. C. Cantereau A. Jegou B. Bourmeyster N. (2018). Phagocytosis by sertoli cells: analysis of main phagocytosis steps by confocal and Electron Microscopy. Methods Mol. Biol. 1748, 85–101. 10.1007/978-1-4939-7698-0_8 29453567

[B368] Yeh J. R. Zhang X. F. Nagano M. C. (2011). Wnt5a is a cell-extrinsic factor that supports self-renewal of mouse spermatogonial stem cells. J. Cell Sci. 124 (14), 2357–2366. 10.1242/jcs.080903 21693582

[B369] Yildirim E. Aksoy S. Onel T. Yaba A. (2020). Gonadal development and sex determination in mouse. Reprod. Biol. 20 (2), 115–126. 10.1016/j.repbio.2020.01.007 32147393

[B370] Yokonishi T. Capel B. (2021). Differentiation of fetal sertoli cells in the adult testis. Reproduction 162 (2), 141–147. 10.1530/Rep-21-0106 34085952 PMC8887120

[B371] Yoshida S. (2020). Mouse spermatogenesis reflects the Unity and diversity of tissue stem cell niche systems. Cold Spring Harb. Perspect. Med. 10 (11), a036186. 10.1101/cshperspect.a036186 32152184 PMC7706566

[B372] You X. Chen Q. Yuan D. Zhang C. C. Zhao H. X. (2021). Common markers of testicular Sertoli cells. Expert Rev. Mol. Diagn. 21 (6), 613–626. 10.1080/14737159.2021.1924060 33945376

[B373] Zaker H. Razi M. Mahmoudian A. Soltanalinejad F. (2022). Boosting effect of testosterone on GDNF expression in Sertoli cell line (TM4); comparison between TM3 cells-produced and exogenous testosterone. Gene 812, 146112. 10.1016/j.gene.2021.146112 34896518

[B374] Zhang Y. Wang S. Wang X. X. Liao S. Y. Wu Y. J. Han C. S. (2012). Endogenously produced FGF2 is essential for the survival and proliferation of cultured mouse spermatogonial stem cells. Cell Res. 22 (4), 773–776. 10.1038/cr.2012.17 22290421 PMC3317562

[B375] Zhang H. J. Yin Y. M. Wang G. S. Liu Z. M. Liu L. Sun F. (2014). Interleukin-6 disrupts blood-testis barrier through inhibiting protein degradation or activating phosphorylated ERK in Sertoli cells. Sci. Rep. 4, 4260. 10.1038/srep04260 24584780 PMC3939460

[B376] Zhang H. L. Chen P. Y. Liu Y. X. Xie W. Q. Fan S. J. Yao Y. C. (2022). Estrogen signaling regulates seasonal changes of the prostate in wild ground squirrels (Spermophilus dauricus). J. Steroid Biochem. Mol. Biol. 218, 106058. 10.1016/j.jsbmb.2022.106058 35017044

[B377] Zhang L. Guo M. Liu Z. Liu R. Zheng Y. Yu T. (2022a). Single-cell RNA-seq analysis of testicular somatic cell development in pigs. J. Genet. Genomics 49 (11), 1016–1028. 10.1016/j.jgg.2022.03.014 35436608

[B378] Zhang W. Mao J. Wang X. Sun B. Zhao Z. Zhang X. (2022b). Case report: novel compound heterozygotic variants in PPP2R3C gene causing syndromic 46, XY gonadal dysgenesis and literature review. Front. Genet. 13, 871328. 10.3389/fgene.2022.871328 35812758 PMC9259967

[B379] Zhang Z. Miao J. Wang Y. (2022c). Mitochondrial regulation in spermatogenesis. Reproduction 163 (4), R55–r69. 10.1530/rep-21-0431 35084362

[B380] Zhang W. Wang X. Mao J. Cao Y. Zhang X. Nie M. (2025). MYRF variants in patients with 46,XY Differences/Disorders of sex development and literature review. Am. J. Med. Genet. A 197 (6), e64002. 10.1002/ajmg.a.64002 39868768

[B381] Zhao L. Koopman P. (2012). SRY protein function in sex determination: thinking outside the box. Chromosome Res. 20 (1), 153–162. 10.1007/s10577-011-9256-x 22161124

[B382] Zhao L. Yao C. Xing X. Jing T. Li P. Zhu Z. (2020). Single-cell analysis of developing and azoospermia human testicles reveals central role of Sertoli cells. Nat. Commun. 11 (1), 5683. 10.1038/s41467-020-19414-4 33173058 PMC7655944

[B383] Zhao X. Wen X. Ji M. Guan X. Chen P. Hao X. (2021). Differentiation of seminiferous tubule-associated stem cells into leydig cell and myoid cell lineages. Mol. Cell Endocrinol. 525, 111179. 10.1016/j.mce.2021.111179 33515640

[B384] Zheng B. Zhao D. Zhang P. Shen C. Guo Y. S. Zhou T. (2015). Quantitative proteomics reveals the essential roles of stromal interaction molecule 1 (STIM1) in the testicular cord Formation in mouse testis. Mol. and Cell. Proteomics 14 (10), 2682–2691. 10.1074/mcp.M115.049569 26199344 PMC4597144

[B385] Zheng W. Nazish J. Wahab F. Khan R. Jiang X. H. Shi Q. H. (2019). DDB1 regulates sertoli cell proliferation and Testis cord remodeling by TGFβ pathway. Genes 10 (12), 974. 10.3390/genes10120974 31779270 PMC6947845

[B386] Zheng G. Y. Chu G. M. Li P. P. He R. (2023). Phenotype and genetic characteristics in 20 Chinese patients with 46,XY disorders of sex development. J. Endocrinol. Invest 46 (8), 1613–1622. 10.1007/s40618-023-02020-8 36745277

[B387] Zhou R. Wu J. R. Z. Liu B. Jiang Y. Q. Chen W. Li J. (2019). The roles and mechanisms of Leydig cells and myoid cells in regulating spermatogenesis. Cell. Mol. Life Sci. 76 (14), 2681–2695. 10.1007/s00018-019-03101-9 30980107 PMC11105226

[B388] Zirkin B. R. Papadopoulos V. (2018). Leydig cells: formation, function, and regulation. Biol. Reprod. 99 (1), 101–111. 10.1093/biolre/ioy059 29566165 PMC6044347

[B389] Zohar-Fux M. Ben-Hamo-Arad A. Arad T. Volin M. Shklyar B. Hakim-Mishnaevski K. (2022). The phagocytic cyst cells in Drosophila testis eliminate germ cell progenitors *via* phagoptosis. Sci. Adv. 8 (24), eabm4937. 10.1126/sciadv.abm4937 35714186 PMC9205596

